# Ambroxol hydrochloride and clenbuterol hydrochloride oral solution for wheezing disorders in children in China: evidence mapping and meta-analysis

**DOI:** 10.3389/fped.2026.1588948

**Published:** 2026-04-22

**Authors:** Yongsheng Guo, Run Guo, Yingxue Zou, Bing Huang, Jiao Li, Mei Yu, Jia Zhai, Shiyin Mu, Yingying An, Weiwei Gao

**Affiliations:** 1 Department of Pulmonology, Machang Campus, Children’s Hospital, Tianjin University/ Tianjin Children’s Hospital, Tianjin, China; 2 Tianjin Key Laboratory of Birth Defects for Prevention and Treatment, Tianjin, China

**Keywords:** evidence mapping, meta-analysis, pediatric patients, randomized controlled trials, respiratory department

## Abstract

**Objective:**

To evaluate the efficacy and safety of ambroxol hydrochloride and clenbuterol hydrochloride oral solution (AHCHOS) in pediatric patients with wheezing disorders and provide a comprehensive evidence map of the current clinical status.

**Methods:**

A literature search was conducted in PubMed, Embase, Cochrane Library, CNKI, Wanfang, and CBM to identify randomized controlled trials (RCTs) evaluating the efficacy and safety of AHCHOS in children with wheezing disorders. The clinical status was descriptively summarized. Meta-analyses for efficacy and safety outcomes were performed using a random-effects model in Review Manager 5.4. Outcomes were treatment effectiveness, times to symptom and clinical sign resolution, length of hospital stay, and adverse events (AEs). All time-related outcomes were reported in days.

**Results:**

A total of 227 RCTs were included in the evidence mapping, 14 of which met the criteria for meta-analysis. Evidence mapping revealed a general increase in the number of studies from 2005 to a peak in 2012, followed by a decline. Most studies were conducted in eastern China, particularly in Henan Province. Meta-analysis results demonstrated that combination therapy with AHCHOS significantly improved the overall response rate [risk ratio (RR) 1.26, 95% confidence interval (CI) 1.10–1.44] and significant effective rate (RR 1.55, 95% CI 1.36–1.76). It also significantly reduced the duration (days) of coughing [mean difference (MD) −1.40, 95% CI −1.75 to −1.06], wheezing (MD −1.88, 95% CI −2.50 to −1.26), cough phlegm (MD −2.00, 95% CI −2.76 to −1.24), wet lung sounds (MD −1.82, 95% CI −2.33 to −1.30), pulmonary rales (MD −2.27, 95% CI −2.90 to −1.64), and hospital stay (MD −1.13, 95% CI −1.45 to −0.82). Furthermore, AHCHOS did not increase the risk of AEs compared with conventional treatment alone.

**Conclusion:**

Combination therapy with AHCHOS was associated with modest but statistically significant improvements in symptom resolution and length of hospital stay compared with conventional treatment alone. However, all included studies were conducted in China, which may limit the generalizability of the findings. In addition, no definitive conclusions regarding safety could be drawn due to limited and inconsistent adverse event reporting. Further safety assessments in future trials are warranted.

**Systematic Review Registration:**

The protocol has been registered on INPLASY (INPLASY202480121).

## 1 Introduction

Wheezing disorders are a prevalent respiratory concern in children, often manifesting during respiratory illnesses such as asthma, bronchiolitis, and pneumonia. Globally, around 50% of infants experience at least one wheezing episode in their first year, and recurrent wheezing is estimated to affect one-third of preschool children (1, 2). The prevalence varies due to factors such as genetic predisposition, nutritional deficiencies, environmental exposures, and a history of allergic disease (3). This condition contributes substantially to morbidity, reduced quality of life, increased healthcare use, school absences, and long-term health issues (2, 4).

Children with wheezing disorders often exhibit a high-pitched, persistent wheeze during respiration, a symptom arising from narrowed airways. This condition can be accompanied by excessive coughing, which may disrupt sleep and reduce the quality of life (5, 6). In infants and young children, excessive airway secretions, rather than sputum production, may contribute to airway obstruction and impaired ventilation (6). Effective airway clearance is therefore an important component in the management of selected respiratory conditions (7).

The management of wheezing disorders in children involves a multifaceted approach tailored to the underlying cause and disease severity (8, 9). These include the use of bronchodilators for rapid symptom relief, inhaled steroids for persistent wheezing, systemic steroids for severe episodes, and antileukotrienes for postviral wheezing, albeit with modest benefits (8). Other treatments, such as antibiotics for confirmed bacterial infections and monoclonal antibodies such as palivizumab for high-risk groups, are used in specific clinical scenarios (8). However, variability in treatment response, potential adverse effects, and adherence challenges necessitate the exploration of alternative or adjunctive therapies (8).

Among these, ambroxol hydrochloride and clenbuterol hydrochloride oral solution (AHCHOS), or Yitanjing (Beijing Hanmi Pharmaceutical Co.,Ltd. No. 10 Tianzhu West Road, Tianzhu Airport Industrial Zone A, Shunyi District, Beijing, China), has gained attention for its dual mechanism of action. Ambroxol may enhance mucociliary clearance by modulating mucus production and promoting ciliary activity, and has been reported to exhibit anti-inflammatory and antioxidant properties (10). Clenbuterol, a β2-adrenergic agonist, may promote bronchodilation and relieve bronchospasm (11) The combination may therefore have additive effects on improving airway obstruction and facilitating mucus clearance.

AHCHOS was approved in China in 2004 for the treatment of acute and chronic respiratory disease. However, its availability in other countries is limited, possibly due to differences in regulatory approval, market demand, or insufficient high-quality clinical evidence to support its widespread use. Therefore, this study aims to provide a comprehensive evidence map and meta-analysis of the clinical status, efficacy, and safety of AHCHOS in the treatment of wheezing disorders in children. By synthesizing the existing data, we provide an overview of the therapeutic benefits and risks of AHCHOS, thereby guiding future clinical practice and contributing to more informed decision-making in the management of pediatric wheezing disorders.

## 2 Methods

This systematic review was conducted following the Preferred Reporting Items for Systematic Reviews and Meta-Analyses (PRISMA) extension for scoping reviews (12) and the PRISMA 2020 statement (13). The protocol has been registered on INPLASY (INPLASY202480121).

### 2.1 Data sources and literature search

We performed electronic database search of PubMed, Embase, Cochrane Library, CNKI, Wanfang, and CBM for eligible randomized controlled trials (RCTs) from inception to May 28, 2024. No restrictions were applied on date, publication status, and language. The search strategy is provided in Supplementary Material S1.

### 2.2 Study selection

To summarize the current clinical status, we included RCTs that examined the efficacy and safety of AHCHOS, used either as a monotherapy or in combination with other treatments, in children under the age of 14 with wheezing disorders, including asthma, bronchiolitis, wheezing bronchitis, mycoplasma pneumonia, and obliterative bronchiolitis. Comparators included other expectorant medications. The outcomes of interest were publication year, geographic location, disease diagnosis, patient age, interventions, and comparators.

To further assess the efficacy and safety of AHCHOS, we selected RCTs that compared the combination of AHCHOS and conventional treatment with conventional treatment alone. Conventional treatment typically includes antiviral and antispasmodic therapy, cough suppression, suctioning, nebulization, and other antiviral treatments. To ensure high-quality evidence, we focused on studies published in Chinese core journals, as listed in *A Guide to the Core Journals of China (2023)*. The primary outcomes included treatment effectiveness, defined by overall response rate, effective rate, and significant effective rate as specified in the original studies. Additionally, we evaluated the time to symptom resolution, including the disappearance of cough, wheezing, and phlegm, as well as the time to clinical sign resolution, including the disappearance of wet lung sounds and pulmonary rales. The outcome “cough phlegm” was defined according to the original studies. It generally reflects clinically assessed airway secretion-related symptoms (e.g., wet cough or auscultatory findings), rather than actual sputum expectoration, particularly in younger children. The length of hospital stay was also considered a primary outcome. All time-related outcomes were reported in days. Secondary outcomes were adverse events (AEs), such as neurological reactions (e.g., headache, drowsiness, insomnia, limb numbness), cardiovascular reactions (e.g., elevated blood pressure, arrhythmias), allergic reactions, and gastrointestinal AEs. Two reviewers independently screened articles for eligibility, with any disagreements resolved through discussion.

### 2.3 Data extraction

Two reviewers independently extracted the data from each study into a predefined data extraction form. For evidence mapping, baseline information was extracted, including publication year, geographic location, disease diagnosis, age, interventions, and comparators. Additionally, more detailed information was extracted for the meta-analysis, including study characteristics (e.g., country, study design), participant characteristics (e.g., sample size, age, and disease diagnosis), treatment characteristics (e.g., treatment regimen, duration, and frequency of administration), and outcomes. Any disagreements between the reviewers were resolved through discussion or with the assistance of a third reviewer.

### 2.4 Data analysis

To qualitatively summarize the clinical status, we used descriptive statistical methods to create an evidence map. This was presented through statistical tables and charts detailing the characteristics of the included trials. To evaluate the efficacy and safety of AHCHOS, a meta-analysis was conducted using Review Manager 5.4. A random-effects model was used to account for variability across studies. For dichotomous outcomes, risk ratio (RR) with a 95% confidence interval (CI) was calculated, while for continuous outcomes, mean difference (MD) with a 95% CI was used to summarize the data. Heterogeneity among studies was assessed using the *I*² statistic and the *χ²* test. Significant heterogeneity was defined as *I*² ≥ 50% and *p* < 0.1 in the *χ*² test (14).

### 2.5 Quality assessment

The quality of the studies included in the meta-analysis was assessed independently by two reviewers using the Cochrane Risk of Bias tool for randomized trials (RoB) (15). Any disagreements were resolved by discussion.

## 3 Results

### 3.1 Study selection results

**Figure 1:**
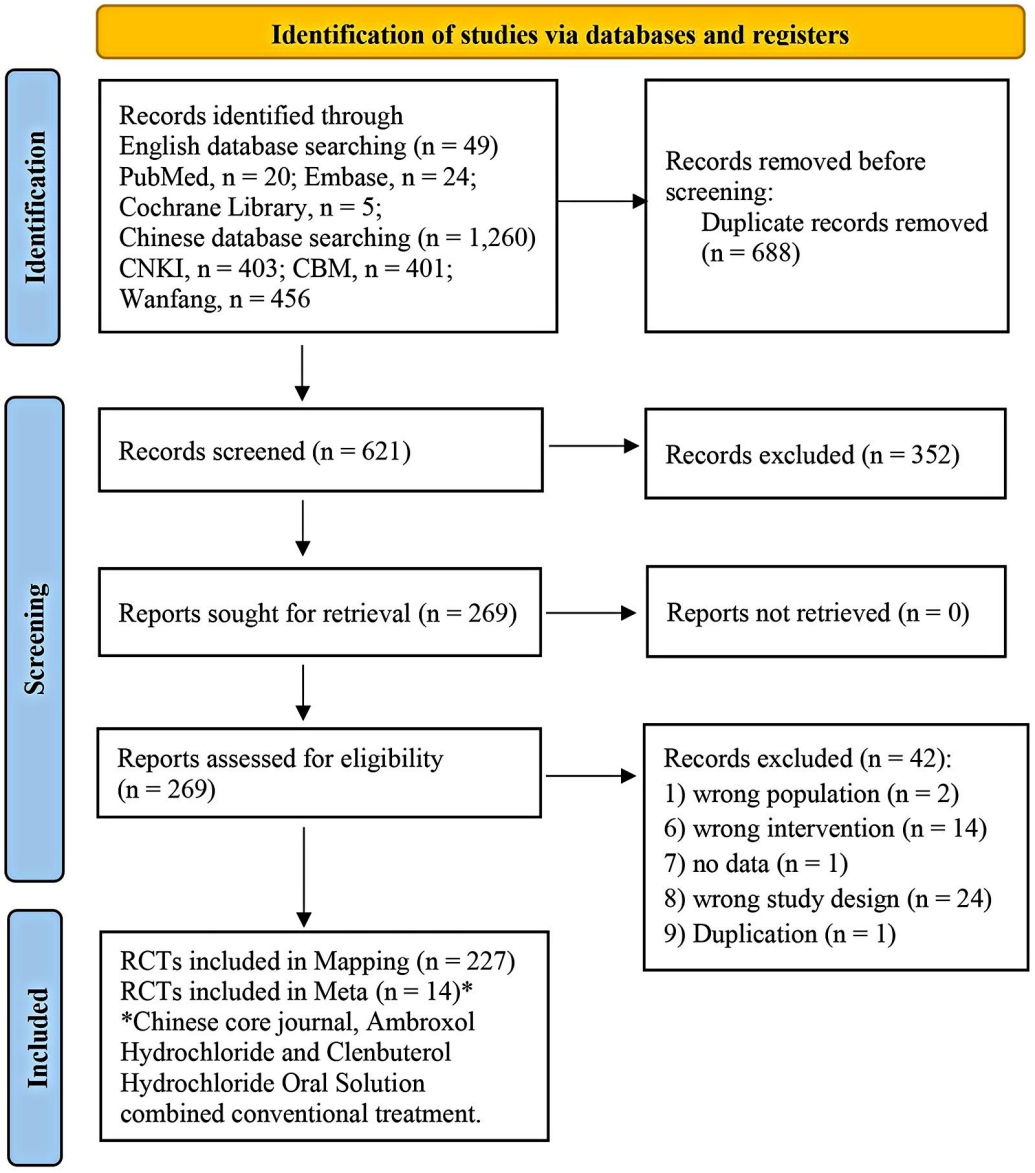
PRISMA study selection flow diagram.

The initial search retrieved a total of 1,309 articles. After removing duplicates, 621 unique articles from the databases remained. Following the screening of titles and abstracts, 269 articles were deemed eligible and retrieved for full-text review. After further screening of full texts, a total of 227 RCTs met the inclusion criteria of the evidence mapping (Supplementary Material S2). Among the 227 RCTs, 14 studies (16–29) that compared AHCHOS and conventional treatment with conventional treatment alone and had been published in Chinese core journals were included in the meta-analysis (Figure 1).

### 3.2 Evidence mapping of clinical status

The earliest identified RCT on AHCHOS was published in 2005. Between 2005 and 2012, the number of studies showed an upward trend that peaked in 2012, which was followed by a decline (Supplementary Figure S1). All 227 RCTs included in the analysis were conducted in China, spanning 31 provinces, municipalities, and autonomous regions. The majority of studies were performed in eastern China, with Henan Province contributing the highest number (32 studies, 14.10% of the total). Collectively, eight provinces—Henan, Guangdong, Shandong, Zhejiang, Shanxi, Guangxi, Liaoning, and Hubei—accounted for 135 studies (59.47% of the total), with each of these provinces contributing at least 10 studies (Supplementary Figure S2).

The total sample size across the 227 RCTs was 27,897 participants, with an average of 123 participants per study. Large-scale studies (≥200 participants) were relatively rare, with only three studies having over 1,000 participants (Supplementary Table S1). The primary objective of all 227 studies was to evaluate the efficacy of AHCHOS. The most frequently conducted comparison (98 studies, 43.17%) was of combined AHCHOS and conventional treatment with conventional treatment alone. The second most common comparison (19 studies, 8.37%) was of combined AHCHOS and conventional treatment with combined ambroxol and conventional treatment (Supplementary Table S2).

All 227 RCTs were assessed as having an unclear risk of bias. While the studies were generally strong in managing incomplete outcome data and selective reporting, there was a significant lack of clarity and transparency in the methods used for random sequence generation, allocation concealment, blinding, and other potential sources of bias (Supplementary Table S3).

### 3.3 Characteristics and quality of studies included in meta-analysis

Fourteen RCTs with 1,741 patients, all published in Chinese core journals, were included in the meta-analysis comparing the combination of AHCHOS and conventional treatment with conventional treatment alone. Patients were diagnosed with bronchiolitis, asthma, pediatric lower respiratory tract infections, pediatric bronchopneumonia, pediatric bronchitis, and severe pneumonia. Among them, three studies involved children and 11 studies were conducted on infants and young children aged 28 days to 3 years (Table 1). All 14 RCTs were rated as having an unclear risk of bias, primarily attributed to selection bias, performance bias, and detection bias (Supplementary Table S4).

**Table 1:** Baseline characteristics of included studies and patients in meta-analysis.

Study ID	Journal	Disease diagnosis	Category	Age (Mean ± SD, months)	Sample size, treatment administration, mode, dose, frequency			
					Combination group		Conventional group	
Zhao 2014	*China Medical Equipment*	Bronchiolitis	Infants	2–24	58	Oral. <8 months: 2.5 mL, bid; 8–12 months: 5 mL, bid; 8 months-2 years: 7.5 mL, bid	48	Conventional treatment such as antiviral antispasmodic, antitussive, aspiration and nebulization
Hou 2013	*Chinese Journal for Clinicians*	Severe pneumonia	Children	Combination group: 27; Conventional group: 31	56	Oral. <12 years: 2.5–15 mL once, bid	56	Oxygen and atomization inhalation, infection control, cough and asthma treatments, prevention of complications
Fang 2013	*China Pharmaceuticals*	Bronchiolitis	Infants	Combination group: 5.36 ± 1.51; Conventional group: 5.31 ± 1.48	30	Oral. 4–8 kg: 2.5 mL, bid; 9–12 kg: 5 mL, bid. Each 100 mL bottle contains 150 mg ambroxol hydrochloride and 100 µg clenbuterol hydrochloride	30	Antiviral treatment, adrenocortical hormone treatment, oxygen-driven atomization inhalation, etc., conventional treatment
Zhang 2012	*Laboratory Medicine and Clinic*	Bronchiolitis	Infants	Combination group: 5.79 ± 1.33; Conventional group: 5.84 ± 1.52	169	Oral. <8 months: 1 mL, bid; 8–12 months: 2 mL, bid; 12–24 months: 3.5 mL, bid	168	Antiviral treatment (antibiotics for bacterial infection), oxygen spray inhalation of budesonide, oxygen inhalation, sputum aspiration, supportive treatment
Yu 2011	*Chinese Pharmacist*	Bronchiolitis	Infants	Combination group: 11; Conventional group: 12	38	Oral. <8 months: 2.5 mL, bid; 8–12 months: 5 mL, bid; 1–2 years: 7.5 mL, bid. Continuous treatment for 7 d. Each milliliter contains 1.5 mg ambroxol hydrochloride and 1 µg clenbuterol	38	All patients treated with ribavirin antiviral therapy, budesonide atomization inhalation, back-patting sputum aspiration, oxygen inhalation, fluid rehydration
Lu 2010	*Journal of Clinical Pediatrics*	Bronchiolitis	Infants	Combination group: 5.39 ± 1.63; Conventional group: 5.34 ± 1.52	48	Oral. <8 months: 2.5 mL, bid; 8–12 months 5 mL, bid; >2 years: 7.5 mL, bid	48	Antiviral treatment (antibiotics for bacterial infection), oxygen spray inhalation of budesonide, oxygen inhalation, sputum aspiration, supportive treatment
Gao 2010	*China Pharmaceuticals*	Bronchiolitis/asthmas	Infants	Combination group: 1.8 ± 0.62 years; Conventional group: 1.8 ± 0.58 years	60	Oral. <8 months: 2.5 mL, bid; 8–12 months: 5 mL, bid; 2–3 years: 7.5 mL, bid	60	Cefuroxime anti-infection treatment, Xiyanping antiviral treatment, rehydration, dexamethasone for anti-inflammation, anti-allergy treatment
Wang 2009	*Guangxi Medical Journal*	Bronchiolitis	Infants	2–24	98	Oral. 4–8 kg: 2.5 mL, tid; 8–12 kg: 5 mL, tid. Continuous treatment for 5–7 d	88	Riba Weilin antiviral treatment, sedation, oxygen inhalation, cough relief, inhalation of atomized *α*-chymotrypsin for expectorant, aminophylline treatment, hydrocortisone for anti-inflammatory treatment, etc.
Zhou 2008	*Journal of Clinical and Experimental Medicine*	Bronchiolitis	Infants	6.3 ± 0.7	100	Oral. <8 months: 2.5 mL, bid; 8–9 months, 5 mL, bid; 1–2 years: 7.5 mL, bid. Continuous treatment for 3–5 d	80	Routine use of cefoperazone, dexamethasone, and ribavirin, inhalation of atomized ribavirin, dihydroxytheophylline as anti-asthmatic, oxygen inhalation, symptomatic treatment
Wang 2008	*Journal of Clinical and Experimental Medicine*	Bronchiolitis	Infants	Not reported	60	Oral. <8 months/4–8 kg: 2.5 mL, bid; 8–12 months/8–12 kg: 5 mL, bid; 2–3 years/12–16 kg: 7.5 mL, bid	60	Anti-infection treatment, cough relief, spasm relief, asthma relief, atomizing inhalation, immunity regulation, sedation, oxygen inhalation, back-patting, expectoration, etc., conventional treatment
Long 2008	*Hainan Medical Journal*	Bronchopneumonia	Infants	Combination group: 1.55 ± 0.46 years; Conventional group: 1.52 ± 0.45 years	50	Oral. <8 months: 2.5 mL, bid; 8–12 months: 5 mL, bid; 2–3 years: 7.5 mL, bid	50	Anti-infection, antispasmodic, and cough treatments, atomization treatment, etc., conventional treatment
Ma 2007	*Journal of Pediatric Pharmacy*	Bronchiolitis	Infants	Not reported	48	Oral. <8 months: 2.5 mL, bid; 8–12 months: 5 mL, bid; 2–3 years: 7.5 mL, bid; 4–5 years: 10 mL, bid; 6–12 years: 15 mL, bid	40	Anti-infection and cough treatments, sputum suction, atomization, etc., conventional treatment, temporary intravenous administration of glucocorticoids in severely ill children
Wang 2006	*Clinical Journal of Medical Officers*	Bronchopneumonia	Children	Not reported	30	Oral. <6 months: 2.5 m, bid; <1 year: 5 mL, bid; <3 years: 7.5 mL, bid; >3 years: 10 mL, bid. Continuous treatment for 5 d. 100 mL contains 150 mg of ambroxol hydrochloride and 100 µg of clenbuterol	30	Routine use of antibiotics (cefuroxime, ceftriaxone, azithromycin, or ribavirin) and fever treatments, oxygen inhalation, other treatment
Huang 2005	*Journal of Pediatric Pharmacy*	Lower respiratory tract infection	Children	Combination group: 20.74 ± 21.76; Conventional group: 20.58 ± 18.40	50	Oral. <8 months: 2.5 mL, bid; 8–12 months: 5 mL, bid; 2–3 years: 7.5 mL, bid; 4–5 years: 10 mL, bid; 6–12 years: 10–15 mL, bid	50	Anti-infection, spasmolytic, and cough treatments, sputum suction, atomization treatment, etc., conventional treatment

### 3.4 Treatment effectiveness

**Figure 2:**
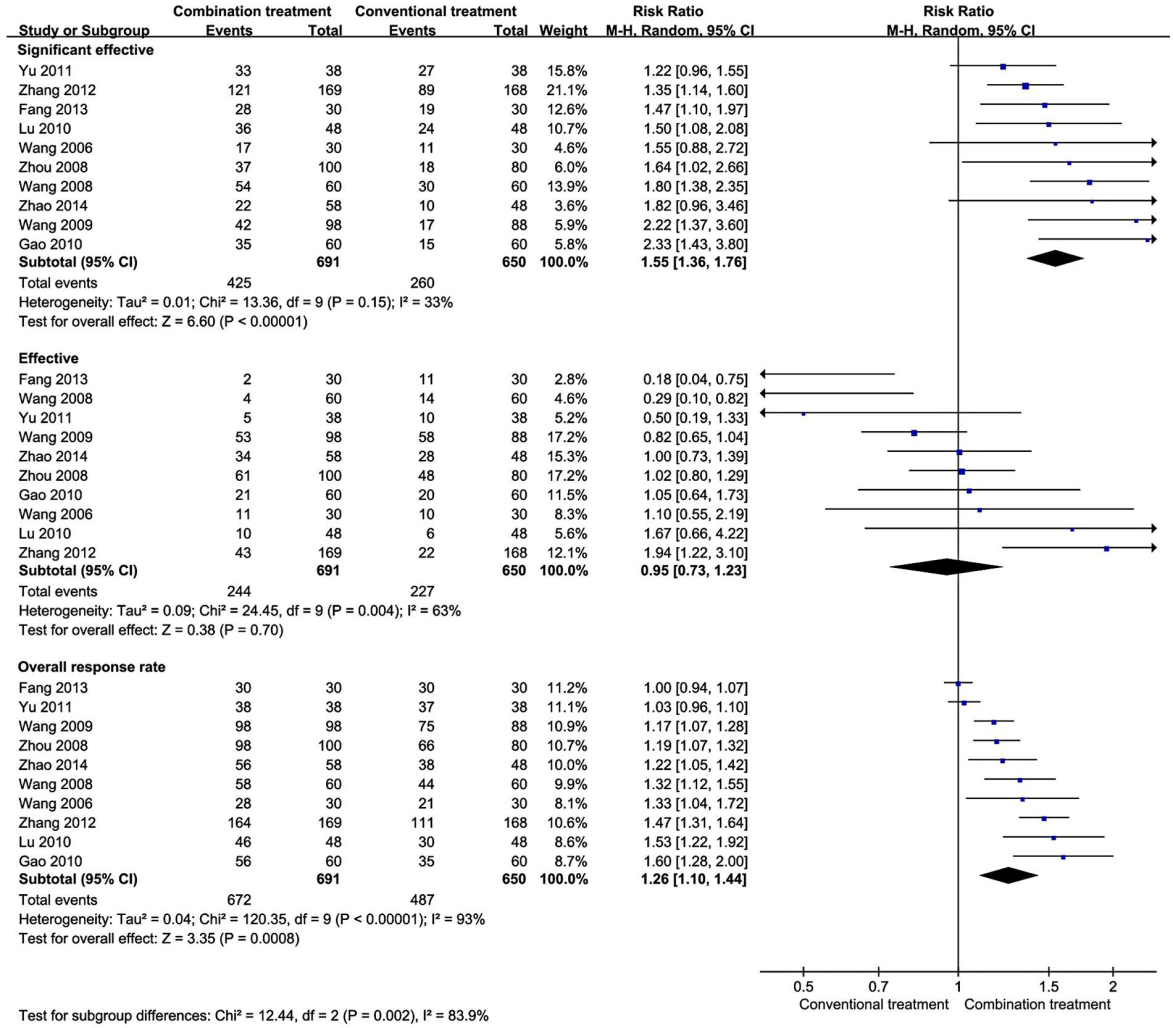
Treatment effectiveness of AHCHOS in the treatment of wheezing disorders in children.

Ten studies with 1,341 patients provided information on treatment effectiveness (Figure 2). The pooled results indicated that the combination of AHCHOS and conventional treatment increased the significant effective rate by 55% compared with conventional treatment alone (RR 1.55, 95% CI 1.36– to 1.76). Similarly, the combined group showed a significantly higher overall response rate than the group with conventional treatment alone (RR 1.26, 95% CI 1.10–1.44). However, no clear difference in the effective rate was observed between the two groups.

### 3.5 Time to symptom resolution

**Figure 3:**
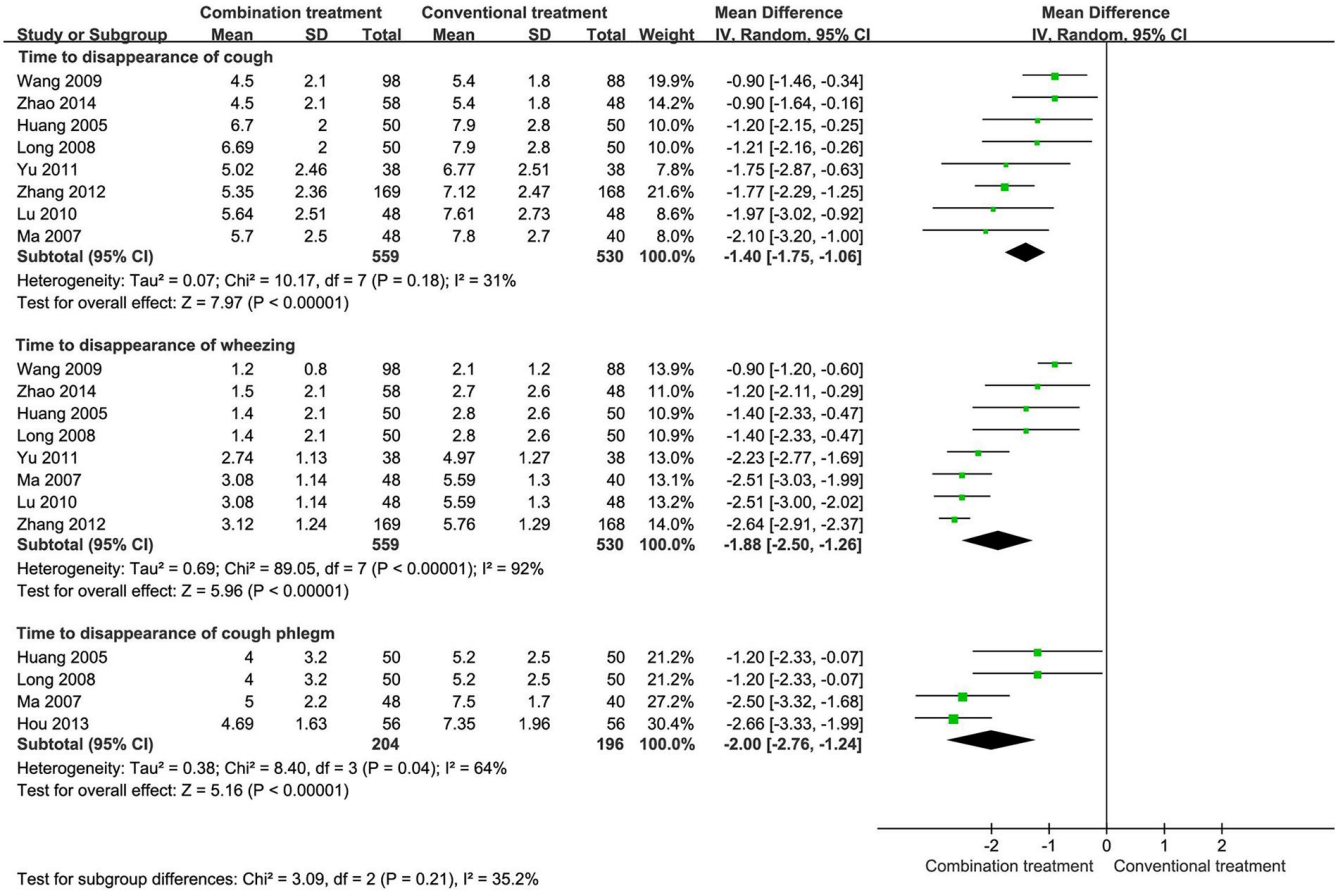
Time to symptom resolution in children with wheezing disorders treated with AHCHOS.

Eight studies with 1,089 patients reported the time to cough and wheeze resolution (Figure 3). The pooled results indicated that the combined group reported a significantly shorter time to cough (MD −1.40, 95% CI −1.75 to −1.06) and wheeze resolution (MD −1.88, 95% CI −2.50 to −1.26) than the group with conventional treatment alone. In addition, four studies with 400 patients reported a significantly shorter duration of cough phlegm in the combined group than in the group with conventional treatment alone (MD −2.00, 95% CI −2.76 to −1.24, Figure 3).

### 3.6 Time to clinical sign resolution

**Figure 4:**
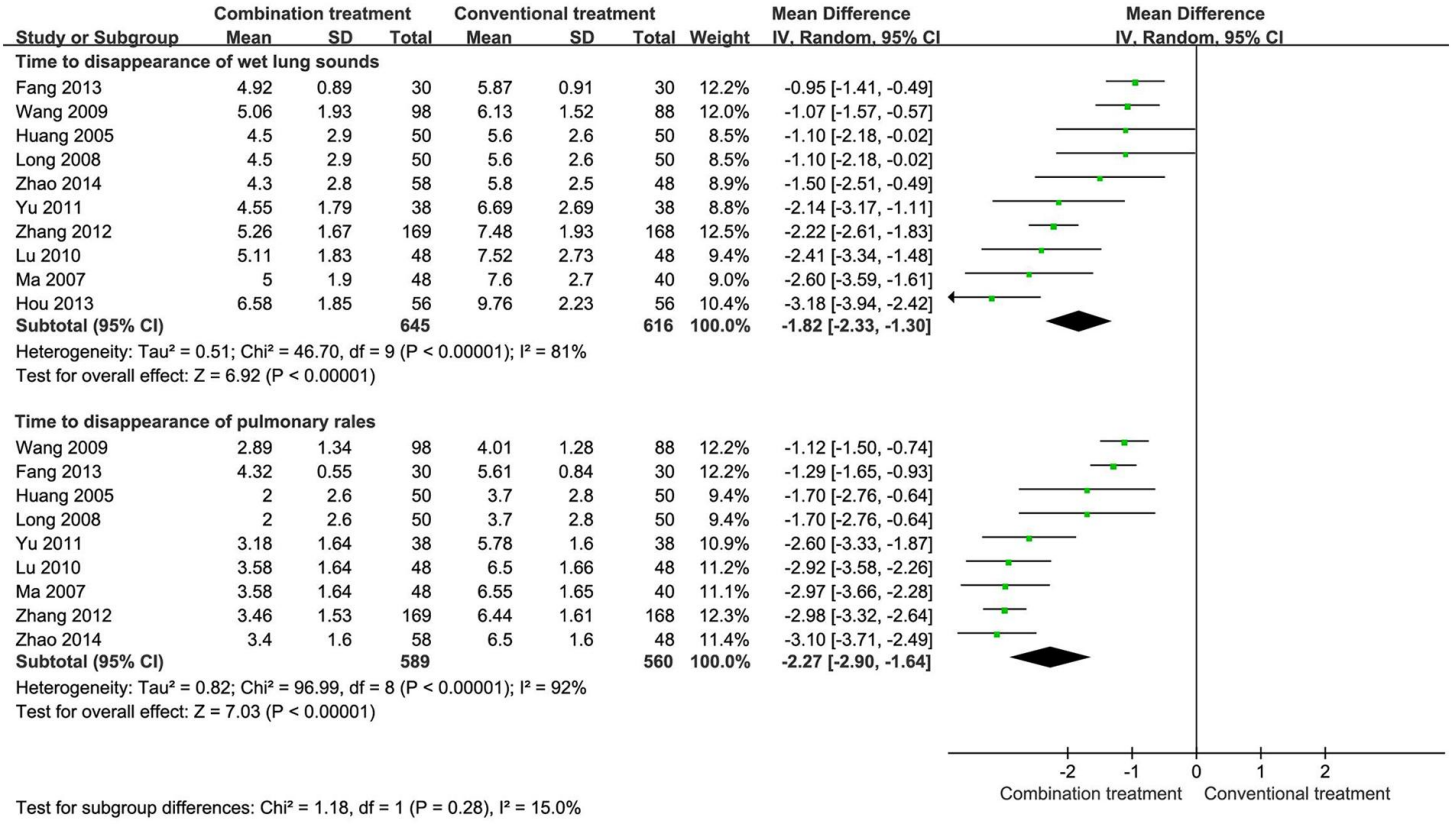
Time to clinical sign resolution in children with wheezing disorders treated with AHCHOS.

The meta-analysis of 10 studies with 1,261 patients demonstrated that the combination of AHCHOS with conventional treatment significantly reduced the time for wet lung sounds to completely dissipate (MD −1.82, 95% CI −2.33 to −1.30, Figure 4). Among these studies, nine studies encompassing 1,149 patients reported on the time to the disappearance of pulmonary rales. The findings consistently showed that the combined treatment led to the more rapid resolution of pulmonary rales than conventional treatment alone (MD −2.27, 95% CI −2.90 to −1.64, Figure 4).

### 3.7 Length of hospital stay

**Figure 5:**
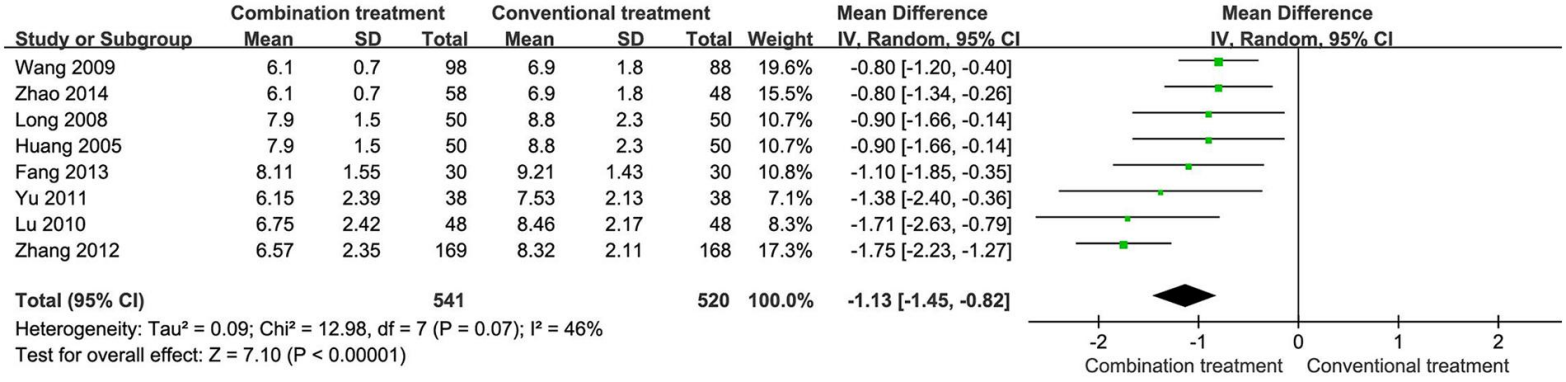
Length of hospital stay for children with wheezing disorders treated with AHCHOS.

The meta-analysis of eight studies with 1,061 patients revealed that the combined treatment group experienced a shorter hospital stay than the group receiving only conventional treatment (MD −1.13, 95% CI −1.45 to −0.82, Figure 5).

### 3.8 Incidence of AEs

**Figure 6:**
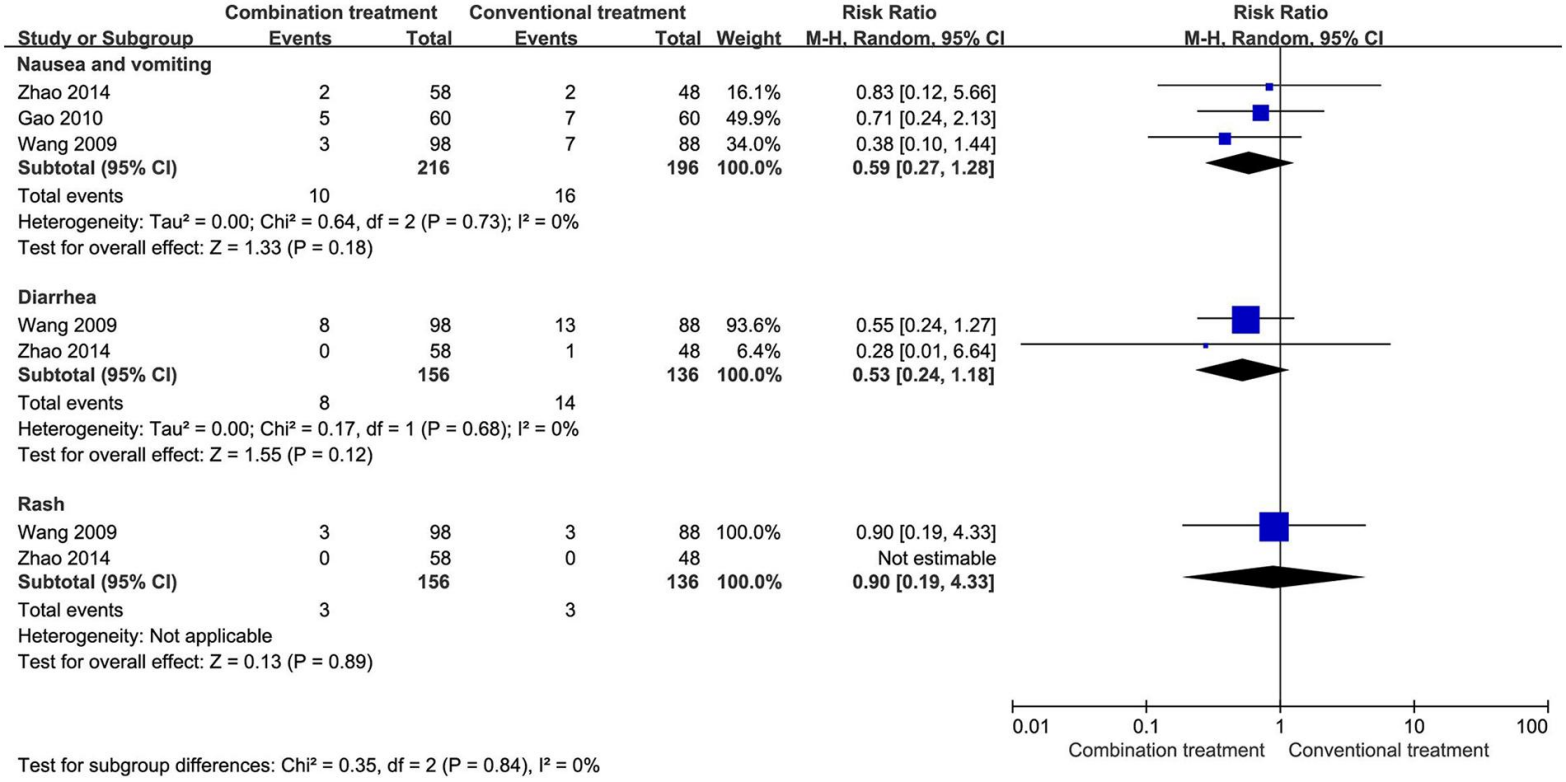
Incidence of AEs in children with wheezing disorders treating AHCHOS.

In a comprehensive review of AEs, three studies involving 412 patients documented the occurrence of nausea and vomiting, while two studies with 292 patients reported the incidence of diarrhea and rash. The pooled analysis of these data did not yield significant differences in the rates of these AEs between the combined treatment group and the group receiving conventional treatment alone (Figure 6).

## 4 Discussion

This systematic review comprehensively summarizes the clinical status and assesses the efficacy and safety of AHCHOS in pediatric patients with wheezing disorders. The findings of the meta-analysis demonstrate that the adjunctive use of AHCHOS with conventional treatment significantly enhances therapeutic outcomes. Specifically, the combination therapy with AHCHOS outperforms conventional treatment in terms of overall response rate and significant effective rate. Moreover, the combination therapy is associated with a shorter time to the resolution of symptoms and clinical signs, as well as a reduced length of hospital stay, compared with conventional treatment alone. In addition, the incidence of AEs did not increase significantly, suggesting a comparable safety profile to that of conventional treatment. Although statistically significant, the observed reductions in symptom duration should be considered in the context of the typically self-limiting nature of pediatric wheezing disorders.

In our synthesis of the clinical landscape, we noted a generally increasing number of AHCHOS-related studies published since 2005, with a notable peak in 2012, after which a decline was observed. This pattern could be linked to the dynamic evolution and continuous refinement of therapeutic approaches for pediatric wheezing disorders (9, 30). Geographic analysis revealed that these studies were exclusively conducted within China, with a pronounced focus on the eastern regions, notably Henan Province. This clustering might be associated with the distribution of medical resources, economic factors, and the allocation of research funding in these areas. Regarding sample size, the cumulative participant count across the studies was 27,897, averaging 123 participants per study, suggesting a predominance of smaller-scale trials. This characteristic could potentially limit the statistical robustness and broader applicability of the findings. The diseases under investigation were predominantly asthma, bronchiolitis, wheezing bronchitis, and pneumonia in children, underscoring the broad potential utility of AHCHOS in managing these prevalent pediatric respiratory conditions. The most common comparison in the studies was between AHCHOS combined with conventional treatment and conventional treatment alone, highlighting the potential of AHCHOS as an adjunctive therapy (30) In addition to comparing AHCHOS with conventional treatments, many studies also explored its combination and comparison with other medications, such as ambroxol (31, 32) and various traditional Chinese medicines (33, 34), suggesting a broad range of potential applications for AHCHOS in pediatric respiratory care.

The results of this meta-analysis on AHCHOS align with previous studies, demonstrating its efficacy in improving therapeutic outcomes for pediatrics with wheezing disorders. For example, a 2011 meta-analysis involving 35 RCTs and 4,545 pediatric patients aged 0–18 years found that the addition of AHCHOS to conventional treatments significantly improved the effective rate (OR 3.98, 95% CI 3.31–4.78) and reduced the duration of symptoms such as cough and wheezing (35). Another meta-analysis published in 2014, comprising 74 RCTs and 8,745 pediatric patients aged 0–12 years, reported comparable findings, with a significant increase in treatment effectiveness when AHCHOS was combined with conventional therapies (OR 3.64, 95% CI 3.18–4.17) (36). Notably, in our analysis, AHCHOS demonstrated a significant improvement in both the overall response rate and the significant effective rate compared with conventional treatments. However, no significant difference was observed in the effective rate. This discrepancy may be attributed to the differing definitions of these outcome measures across the included studies. In most trials, “significant effective” referred to the complete disappearance of cough, wheezing, dyspnea, and abnormal lung sounds after 6–7 days of treatment, whereas “effective” denoted only partial improvement in these symptoms during the same period. The overall response rate was typically calculated as the sum of these two categories. Because the assessment of partial improvement is more subjective and prone to variation across clinicians and study settings, misclassification is likely, which may obscure between-group differences in the “effective” category. This heterogeneity in outcome definitions constitutes an important methodological limitation and underscores the need for standardized criteria when comparing treatment effects across studies. Moreover, differences in disease severity may lead to different treatment responses among patients. Namely, patients with milder conditions may show rapid improvement, increasing the overall effective rate or significant effective rate, whereas patients with more severe conditions may not meet the criteria for significant improvement, thereby reducing the effective rate.

In addition to improving clinical outcomes, the pharmacological action of AHCHOS may contribute to the observed symptom (37, 38). Ambroxol hydrochloride acts on airway secretory cells by modulating the secretion of serous and mucous glands, increasing serous secretion, and reducing sputum viscosity. It can also restore the activity of bronchial epithelial cells, enlarge the functional space of ciliary movement, and enhance both the frequency and intensity of ciliary beating, thereby facilitating sputum clearance. In addition, ambroxol stimulates type II alveolar epithelial cells to synthesize and secrete pulmonary surfactant, which helps maintain alveolar stability and distal airway patency, exerts anti-adhesive effects, and promotes mucus transport. Clenbuterol hydrochloride is a highly selective β-adrenergic receptor agonist that effectively relieves bronchospasm and enhances the movement of respiratory tract cilia (39). These combined mechanisms may synergistically alleviate airway obstruction, improve mucociliary clearance, and accelerate symptom resolution.

In our analysis, AHCHOS also demonstrated a significant reduction in the duration of symptoms and signs such as cough, wheezing, cough phlegm, wet lung sounds, and pulmonary rales, consistent with previous research (35, 36). Although the reductions in symptom and sign durations were modest in absolute terms, they are clinically meaningful in pediatric practice. The combined therapy shortened cough duration by approximately 1.4 days and wheeze resolution by nearly 2 days, improvements that can substantially relieve symptom burden, enhance sleep and feeding, and reduce caregiver stress (4). Similarly, the 1.8–2.3 day acceleration in the resolution of wet lung sound and the 2-day reduction in cough phlegm duration suggest faster recovery of airway patency and mucociliary function. Moreover, shorter hospitalization times decrease both disease and caregiving burdens, enabling a quicker return to normal activities and potential healthcare cost savings. These findings highlight the real-world clinical and economic advantages of incorporating AHCHOS into treatment for pediatric respiratory conditions.

Regarding the safety profiles of AHCHOS, the available evidence is limited. Only a minority of included studies reported no significant difference in the incidence of AEs such as nausea, vomiting, diarrhea, and rash between the group receiving the combination therapy and the group on conventional treatment alone. However, the small number of reporting studies and the restricted sample size substantially limit the robustness of these findings. Moreover, none of the trials monitored or reported clinically important potential adverse effects associated with β2-agonists, such as tachycardia, palpitations, or other cardiovascular events. This lack of systematic safety assessment precludes a comprehensive evaluation of the safety profile of AHCHOS. Therefore, the safety-related conclusions of this review should be interpreted with caution. Future randomized controlled trials should incorporate standardized and thorough adverse event reporting, with particular attention to cardiovascular outcomes, to more accurately establish the safety of AHCHOS in pediatric populations.

Beyond efficacy and safety, AHCHOS may offer additional advantages in clinical application, particularly in younger paediatric populations. Respiratory tract infections are highly prevalent among children and are often accompanied by viscous sputum and wheezing. As a compound preparation combining ambroxol hydrochloride and clenbuterol hydrochloride, AHCHOS simplifies treatment regimens by reducing the number of separate medications required. This can improve the convenience of administration for caregivers and enhance medication adherence in young children, who often present greater challenges in treatment compliance due to age-related limitations. Such advantages may further support the clinical utility of AHCHOS in real-world paediatric practice, especially in age groups where adherence to complex medication schedules is difficult. Apart from these practical considerations, the pharmacological properties of the two active components may also help explain the clinical benefits observed. Ambroxol enhances mucociliary clearance, reduces sputum viscosity, and exerts anti-inflammatory effects, whereas clenbuterol provides bronchodilation through β2-adrenergic stimulation. These complementary actions may work synergistically to improve airway patency and secretion clearance, thereby accelerating symptom resolution. Nevertheless, direct evidence confirming this synergistic interaction remains limited, and further mechanistic studies are needed to elucidate the combined therapeutic effect.

It is well-noted that Oral β2-adrenergic agonists have largely been abandoned in many international guidelines due to concerns regarding limited efficacy and increased systemic adverse effects, with inhaled therapies now preferred. However, such agents remain widely used in China, possibly due to historical prescribing practices, differences in healthcare settings, and the convenience of oral administration, particularly in pediatric populations. Therefore, the findings of this study should be interpreted within this specific clinical context. While the results suggest potential benefits, their applicability to regions where oral β2-agonists are not routinely used may be limited.

One of the strengths of our study is the use of evidence mapping to provide a comprehensive overview of the clinical landscape, thereby highlighting research gaps and offering valuable insights to guide future clinical studies. Additionally, we presented updated data on the efficacy and safety of AHCHOS through a meta-analysis, contributing to a more current understanding of its clinical benefits. Our study also adhered to strict inclusion criteria based on *A Guide to the Core Journals of China (2023)*, which resulted in fewer eligible trials than in previous meta-analyses and ensured greater quality control, thus enhancing the reliability of our results.

However, our study had some limitations. Firstly, although we performed a comprehensive search of both English- and Chinese-language databases, all eligible studies ultimately originated from the Chinese-language literature. This restricts the generalizability of our findings to pediatric populations outside mainland China and highlights a current gap in international research on AHCHOS. This regional concentration of studies may be partly attributable to the fact that AHCHOS is a compound formulation primarily developed and marketed by Chinese pharmaceutical manufacturers, with regulatory approval and routine clinical use largely confined to China. In addition, clenbuterol, as a β2-adrenergic agonist included in AHCHOS, is approved for respiratory indications in China and certain Asian countries but is highly restricted or prohibited in many European and North American regions because of safety considerations, limiting related research in those settings. Although ambroxol is widely used globally, its combination with clenbuterol has not been widely adopted outside China. These factors collectively contribute to the geographical specificity of the existing RCTs and should be considered when interpreting the external validity of our findings. Secondly, most of the studies included in the meta-analysis primarily involved infants and toddlers aged 0–3 years, with limited research on older children aged 4–12 years and 13–18 years. Consequently, the relevance of the findings to these age groups is limited, and further clinical trials are needed to assess the efficacy and safety of AHCHOS for a broader age range. Additionally, the majority of studies focused on bronchitis, limiting the applicability of the findings to other conditions, such as pneumonia and asthma. More rigorous studies are needed to assess the efficacy and safety of AHCHOS for these diseases. Also, the variability in the outcome definitions across the included studies introduced heterogeneity, as we adopted the original definitions of treatment effectiveness from each study. The data are also limited by the lack of clarity in outcome definitions.

In addition, all studies were assessed as having an unclear risk of bias, particularly concerning random sequence generation, allocation concealment, and blinding. These methodological issues may overestimate treatment effects and could potentially affect the reliability of the results. When considered against key evidence appraisal domains, the overall certainty of the evidence is therefore likely limited, despite the consistency of findings. This underscores the need for future trials to adopt rigorous randomization, concealment, and blinding procedures, to strengthen the quality of evidence. Furthermore, although the evidence map identified several studies comparing AHCHOS plus conventional therapy with ambroxol-based regimens, substantial heterogeneity in dosing and outcome definitions made quantitative synthesis infeasible. Therefore, our meta-analysis focused on AHCHOS plus conventional therapy vs. conventional therapy alone. This highlights an important evidence gap, and future well-designed head-to-head trials comparing AHCHOS with ambroxol monotherapy using standardized dosing protocols and outcome measures are needed to clarify the added clinical value of AHCHOS.

## 5 Conclusion

This systematic review and meta-analysis indicate that, under the current evidence base, which predominantly derived from studies conducted in China, the combination of AHCHOS with conventional treatment may improve clinical outcomes in infants with bronchitis and related wheezing disorders. The combination appears to shorten key symptom and sign durations and reduce hospital stay without increasing AEs. However, the absolute magnitude of benefit was relatively modest, with reduction in symptom duration generally ranging from approximately 1–2 days. These findings suggest potential clinical and practical benefits, particularly in improving patient comfort and reducing caregiver burden among young children in China. Given the moderate methodological quality, limited geographic representation, and narrow age range of the included studies, these findings should be interpreted with caution. While the observed improvements may offer practical advantages in terms of symptom relief and reduced hospitalization, they may not necessarily translate into substantial clinical benefit in all settings. High-quality, multicenter international randomized controlled trials that include broader age groups and diverse populations are needed to further establish the effectiveness and safety of AHCHOS in pediatric respiratory care.

## Data Availability

The original contributions presented in the study are included in the article/Supplementary Material, further inquiries can be directed to the corresponding author.
