# Supplementary material for: Ambroxol hydrochloride and clenbuterol hydrochloride oral solution for wheezing disorders in children in China: evidence mapping and meta-analysis
Source: Front Pediatr. 2026 Apr 22;14:1588948. doi: 10.3389/fped.2026.1588948 (PMC13143944; doi:10.3389/fped.2026.1588948)
Supplement: Supplementary file 1 [file Datasheet1.pdf]

Supplementary files

Contents

Supplementary 1 Search Strategy 2

Supplementary 2 Reference list of 227 studies included in the evidence mapping 4

Supplementary 3 Evidence mapping results 13

Supplementary 4 Meta-analysis results 27

Supplementary 1 Search Strategy

2024-5-28

PubMed

"chf 023"[tw] OR chf023[tw] OR clembroxol[tw] OR (clenbuterol[tw] AND ambroxol[tw]) OR yitanjing[tw] OR ventoliber[tw] 20

Embase

'ambroxol plus clenbuterol'/exp OR ("chf 023" OR chf023 OR clembroxol OR (clenbuterol NEAR/2 ambroxol) OR yitanjing OR ventoliber):ab,ti,kw 24

Cochrane Library

("chf 023" OR chf023 OR clembroxol OR (clenbuterol NEAR/2 ambroxol) OR yitanjing OR ventoliber):ab,ti,kw 5

CNKI（期刊、学位、会议，中英文扩展：是，中文）

(SU%=氨溴特罗+氨溴索*克仑特罗+易坦静 OR TKA % 氨溴特罗+氨溴索*克仑特罗+易坦静) AND (SU%=儿童+婴儿+幼儿+婴幼儿+少儿+小儿+学龄+学龄前+学生+小学生+早产儿+新生儿+儿科+低龄+适龄+患儿+学龄+小学生+中学生+学生+青少年+少年 OR TKA % 儿童+婴儿+幼儿+婴幼儿+少儿+小儿+学龄+学龄前+学生+小学生+早产儿+新生儿+儿科+低龄+适龄+患儿+学龄+小学生+中学生+学生+青少年+少年) AND (SU%=哮喘+喘息性疾病+喘息性支气管炎+喘支+喘息性+喘息型+喘+咳喘性+咳喘型+哮鸣音+气道炎症+毛细支气管炎+支原体肺炎+支气管肺炎 OR TKA % 哮喘+喘息性疾病+喘息性支气管炎+喘支+喘息性+喘息型+喘+咳喘性+咳喘型+哮鸣音+气道炎症+毛细支气管炎+支原体肺炎+支气管肺炎)
403

万方（期刊、学位、会议）

(主题:("氨溴索" AND "克仑特罗") or 主题:("氨溴特罗" OR "易坦静")) and 主题:(儿童 OR 婴儿 OR 幼儿 OR 婴幼儿 OR 少儿 OR 小儿 OR 学龄 OR 学龄前 OR 学生 OR 小学生 OR 早产儿 OR 新生儿 OR 儿科 OR 低龄 OR 适龄 OR 患儿 OR 学龄 OR 小学生 OR 中学生 OR 学生 OR 青少年 OR 少年) and 主题:("哮喘" OR "喘息" OR "喘支" OR "喘" OR "咳喘" OR "哮鸣音" OR "气道炎症" OR "毛细支气管炎" OR "支原体肺炎" OR "支气管肺炎") 456

CBM

(("氨溴索"[常用字段:智能] AND "克仑特罗"[常用字段:智能]) OR "氨溴特罗"[常用字段:智能] OR "易坦静"[常用字段:智能]) AND ("儿童"[常用字段:智能] OR "婴儿"[常用字段:智能] OR "幼儿"[常用字段:智能] OR "婴幼儿"[常用字段:智能] OR "少儿"[常用字段:智能] OR "小儿"[常用字段:智能] OR "学龄"[常用字段:智能] OR "学龄前"[常用字段:智能] OR "学生"[常用字段:智能] OR "小学生"[常用字段:智能] OR "早产儿

"[常用字段:智能] OR "新生儿"[常用字段:智能] OR "儿科"[常用字段:智能] OR "低龄"[常用字段:智能] OR "适龄"[常用字段:智能] OR "患儿"[常用字段:智能] OR "学龄"[常用字段:智能] OR "小学生"[常用字段:智能] OR "中学生"[常用字段:智能] OR "学生"[常用字段:智能] OR "青少年"[常用字段:智能] OR "少年"[常用字段:智能]) AND ("哮喘"[常用字段:智能] OR "喘息性疾病"[常用字段:智能] OR "喘息性支气管炎"[常用字段:智能] OR "喘支"[常用字段:智能] OR "喘息性"[常用字段:智能] OR "喘息型"[常用字段:智能] OR "喘"[常用字段:智能] OR "咳喘性"[常用字段:智能] OR "咳喘型"[常用字段:智能] OR "哮鸣音"[常用字段:智能] OR "气道炎症"[常用字段:智能] OR "毛细支气管炎"[常用字段:智能] OR "支原体肺炎"[常用字段:智能] OR "支气管肺炎"[常用字段:智能]) 401

Supplementary 2 Reference list of 227 studies included in the evidence mapping

1. AN ZH. Observation of the treatment effect of ambroxol and clenbuterol oral solution for acute lower respiratory tract infection of children. Journal of Foshan University (Natural Science Edition). 2008;26(3):64-5.
2. BAI XL, XI XH, SHI HY, WANG FQ. Curative effect of ambroterol in the adjuvant treatment of bronchiolitis. China Clinical Practical Medicine. 2010;04(7):183.
3. BAI YS. Clinical observation of ambroterol oral solution in treatment of bronchiolitis in children. Chinese Journal of Modern Drug Application. 2019;13(19):70-1.
4. BI SF. Therapeutic effect of oral medication combined with budesonide and ipratropium bromide in the treatment of children with bronchiolitis. China Practical Medicine. 2019;14(25):79-80.
5. BU YZ. Analysis of clinical efficacy of ambroterol oral liquid in improving respiratory symptoms in children with bronchitis. Medicine and Human. 2014;27(10).
6. CAI JM. Clinical efficacy and safety of ambroxol combined with compound ipratropium bromide and terbutaline in the treatment of bronchitis in children. Contemporary Medicine. 2020;26(01):24-6.
7. CAI ZH, YANG QL, LI XD. Study on the efficacy of Singulair combined with ethanazine in the treatment of bronchiolitis. Guide of China Medicine. 2016;14(29):63-4.
8. CAO B. Clinical effect of ambroterol oral liquid on children with asthma complicated with respiratory tract infection. Chinese Journal of Modern Drug Application. 2010;4(10):125-6.
9. CAO XL, LI LL, GENG RJ, BU Q, CUI Y. Effect of ambroterol combined with cefaclor on levels of chitinase-like protein-40 and cysteinotriene in children with acute bronchitis. Maternal and Child Health Care of China. 2023;38(20):3918-21.
10. CEN YM. Clinical effect of ambroterol combined with compound ipratropium bromide in the adjuvant treatment of pediatric pneumonia. Chinese Journal of Clinical Rational Drug Use. 2020;13(28):24-5+7.
11. CHEN CL, WANG MY, XU B. Study on Effect of Hydrochloride Oral Solution Combined with Bromhexine in Treatment of Pedo-bronchopneumonia. Clinical Medical & Engineering. 2010;17(07):61-2.
12. CHEN GJ. Curative effect of ambroterol oral liquid on lower respiratory tract infection in children. Medical Innovation of China. 2009;6(19):63-4.
13. CHEN LQ. Observation of curative effect of ambroterol oral liquid on children with asthmatic diseases. National Medical Frontiers of China. 2013;8(06):56+2.
14. CHEN MG. Curative effect of ambroterol in the adjuvant treatment of bronchiolitis in children. Guide of China Medicine. 2011;9(16):250-1.
15. CHEN QY. Clinical analysis of ambroterol oral solution in the treatment of acute respiratory tract infection in children. Health Care Today 2015(12):67-8.
16. CHEN SQ. Clinical study on the application of ambroterol oral liquid in the treatment of bronchitis in children. Inner Mongolia Medical Journal. 2019;51(9):1066-8.
17. CHEN Y, WANG CX, LIU Y. Observation on the clinical effect of Yi Tanjing in the treatment of acute respiratory tract infection in children. Guide of China Medicine. 2013;11(05):456-7.
18. CHEN YM. Analysis of clinical value of ambroterol oral liquid in the treatment of bronchopenitis in children. Guide of China Medicine. 2014;12(11):103-4.
19. DENG LF. Clinical effect of ambroterol oral liquid in adjuvant treatment of asthmatic bronchitis in children. Health for all. 2017(18):12.
20. DENG TJ, WANG P. Analysis of therapeutic effect of ambroterol oral liquid on bronchitis in children. scientific and technological innovationInformation. 2011(19):20.
21. DENG YJ. Therapeutic effect of ambroterol oral liquid combined with Montelukast adjuvant in treating 40 cases of bronchiolitis. Good Health for All. 2016;10(03):154-5.
22. DING BX. Analysis of effect of ammonia bromine Trow oral in treatment of babies and infants with bronchopneumonia. Journal of Medical Forum. 2012;33(04):57-8.
23. DONG JW. Therapeutic effect of ambroterol oral liquid in the treatment of bronchiolitis. Journal of Modern Medicine & Health. 2008(22):3369.
24. DU DL. Observation on curative effect of ambroterol oral liquid in treatment of bronchiolitis. Good Health for All. 2013;7(09):67-8.
25. DUAN CY. Ambroterol combined with Montelukast for treatment of 60 cases of asthma in children. Shenzhen Journal of Integrated Traditional Chinese and Western Medicine. 2015;25(12):103-4.
26. FAN H, XU JX, HUANG J, ZHANG XX, HU AX. Clinical Study on 40 Children Bronchial Asthma Cases Treated by Maxin Pingchuan Decoction. Journal of Traditional Chinese Medicine. 2011;52(13):1112-4+8.
27. FANG XY, LU HH. Ambroterol oral solution for treatment of 30 cases of bronchiolitis. China Pharmaceuticals. 2013;22(05):97-8.

28. FENG HN. Efficacy of azithromycin combined with ambroterol in the treatment of mycoplasma pneumonia in children. Shenzhen Journal of Integrated Traditional Chinese and Western Medicine. 2015;25(18):148-9.
29. FU H. Efficacy evaluation of azithromycin combined with ambroterol in treatment of mycoplasma pneumonia in children. Guide of China Medicine. 2012;10(27):582-3.
30. GAO CX, SHEN JH, CHEN Y. Observation on the curative effect of ambroterol oral solution on relieving cough, expelling phlegm and relieving asthma. China Pharmaceuticals. 2010;19(17):75-6.
31. GAO XP, HAO RQ, HAO LM. Application of Yi Tanjing in the treatment of mycoplasma pneumonia in children. Proceedings of the 5th Chinese Pharmacists Conference; 杭州 2013. p. 1-3.
32. GONG JL. Clinical effect of ambroterol on children with bronchiolitis. Guide of China Medicine. 2012;10(01):73-4.
33. GU MF, ZHANG JM. Clinical application of ambroterol oral liquid in the treatment of mycoplasma pneumonia in children. Chinese Primary Health Care. 2013;27(03):41-2.
34. GUAN AL. Observation of curative effect of clenbuterol hydrochloride combined with compound ipratropium bromide in the treatment of children with bronchiolitis. Journal of Clinical Pulmonary Medicine. 2014;19(02):206-8.
35. GUO CL. Evaluation of therapeutic effect of ambroterol oral liquid and azithromycin on 83 cases of mycoplasma pneumonia in children. Journal of Medical Forum. 2011;32(11):137-8.
36. GUO FL, HE JN, CHEN SL. Bronchiolitis treatment by ambroxol hydrochloride and clenbuterol hydrochloride oral solution. Chinese Pediatric Emergency Medicine. 2007;14(04):338-9.
37. GUO H, BAI L, LUO XH. Analysis of the Effect of Yitanjing Adjuvant Treatment of Infantile Viral Pneumonia. Medical Information. 2014(17):78-.
38. GUO HL. Curative effect of ambroterol oral liquid combined with Montelukast in treating 35 cases of bronchiolitis. Journal of Aerospace Medicine. 2012;23(12):1483-4.
39. GUO LJ. Analysis of curative effect of ambroterol oral liquid on mycoplasma pneumoniae pneumonia in children. Yiyao Qianyan. 2016;6(13):117-8.
40. GUO Q. Analysis of clinical efficacy of Yi Tanjing in the adjuvant treatment of bronchopneumonia in children. World Health Digest 2013(3):189-.
41. GUO XH. Study on Clinical Effect of Ambroxol Combined With Compound Ipratropium Bromide in the Treatment of Children With Bronchiolitis. China Continuing Medical Education. 2016;8(12):124-5.
42. HE J, ZHAO XQ, QIU JM. Therapeutic effect of ambroterol on 43 cases of acute bronchiolitis. Guizhou Medical Journal. 2007;31(11):1025-.
43. HE JF. Curative effect of ambroterol oral liquid on asthmatic bronchitis. China Practical Medicine. 2011;6(20):139-40.
44. HE M. Curative effect of ambroterol oral liquid in adjuvant treatment of asthmatic diseases in children. Shenzhen Journal of Integrated Traditional Chinese and Western Medicine. 2020;30(16):101-2.
45. HONG JG, LI YZ, LU Q, ZHANG LE, WANG LB, LI Z, et al. Clinical observation of ambroterol oral liquid in improving respiratory symptoms in children with bronchitis. Chinese Journal of Practical Pediatrics. 2006(01):63-5.
46. HOU CM. Observation on the curative effect of ambroteroxor in treating 56 cases of severe pneumonia in children. Chinese Journal for Clinicians. 2013;41(02):57-8.
47. HU FW. Therapeutic effect of ambroterol oral liquid on bronchopneumonia in children. Road to Health Magazines. 2015(2):61-,2.
48. HU XJ, WU JG. Effect of ambroterol on 61 cases of lower respiratory tract infection in infants. Journal of Community Medicine. 2015;13(02):52-3.
49. HU XJ, WU XP. Clinical efficacy of ambroterol oral solution combined with Montelukast in the treatment of bronchiolitis. China Health Care & Nutrition. 2017;27(33):308.
50. HUANG CM. Clinical observation of ambroterol oral liquid in adjuvant treatment of bronchiolitis. Guide of China Medicine. 2017;15(08):191.
51. HUANG RJ. Clinical analysis of ambroterol hydrochloride oral solution for treatment of 132 cases of infant bronchiolitis. Seek Medical and Ask the Medicine. 2012;10(06):509.
52. HUANG Y, JIANG HZ, YAN L, LI QB, LUO ZX, LUO J. Observation on efficacy of Yitanjing on children with lower respiratory tract infection. Journal of Pediatric Pharmacy 2005;11(6):18-9.
53. HUO JF. Therapeutic effect of ambroterol oral solution combined with compound ipratropium bromide solution on bronchiolitis. Hebei Journal of Traditional Chinese Medicine. 2011;33(08):1276-7.
54. JIA CF. Observation of Infant Bronchliolitis Treated with Ambroxol Hydrochloride and Clenbuterol Hydrochloride Oral Solution and Traditional Chinese Medicine Nursing. Chinese Medicine Modern Distance Education of China. 2013;11(16):146-7.
55. JIA YT. Analysis of curative effect of ambroterol oral liquid on children with asthmatic diseases. Journal of Medical Forum. 2013;34(07):107-8.

56. JIANG HZ, DAI HY. Observation on the effect of Yitanjing in the adjuvant treatment of lower respiratory tract infection in children. Chinese Health Care. 2007;15(2):29-.
57. JIAO HF, SHI QJ. Ambroxol hydrochloride and clenbuterol hydrochloride oral solution in the treatment of infants with bronchiolitis. Chinese Journal of Practical Medicine. 2012;39(4):10-1.
58. KANG PP, ZHANG XZ, CHEN XW. Effects of Ambroxol hydrochloride and Clenbuterol hydrochloride combined with Budesonide in treatment of children with acute bronchitis. Medical Journal of Chinese People's Health. 2023;35(15):59-61,5.
59. LENG F, LI P. Therapeutic effect of ambroterol oral liquid combined with azithromycin on 62 cases of mycoplasma pneumonia in children. Chinese Community Doctors. 2011;13(22):141.
60. LI DC. Clinical effect of ambroterol oral liquid on bronchitis in children and its influence on clinical symptoms. China Health Care & Nutrition. 2021;31(33):195.
61. LI DJ. Curative effect of ambroterol on lower respiratory tract infection in infants. Proceeding of Clinical Medicine. 2009;18(10):685-6.
62. LI DL, LI CR, ZHONG GQ. Effect of Interferon and Yitanjing on Infants with Bronchiolitis. Medical Innovation of China. 2012;9(21):30-1.
63. LI H, HAN PQ, WANG RZ. Therapeutic effect of ambroterol oral liquid on 89 cases of bronchophenitis in infants. Journal of Shanxi College of Traditional Chinese Medicine. 2010;11(06):66-7.
64. LI ND, WU YP, LIAO HC. Effect of ambroxol oral liquid on recovery time and prognosis of children with acute bronchitis. China Medicine and Pharmacy. 2017;7(24):61-3.
65. LI SF. Clinical efficacy of ambroterol oral liquid in the treatment of children with bronchitis. Electronic Journal of Clinical Medical Literature. 2018;5(46):149,60.
66. LI SY. Curative effect of ambroterozox on 38 cases of bronchiolitis in children. Good Health for All. 2010;26(8):5-7.
67. LI XF, HUANG RC, LI SL, CAO ZP, LI WX, LI JH. Therapeutic effect of ambroterol oral liquid on asthmatic bronchopneumonia in infants. Medicine and Human. 2014;27(06):74.
68. LI XJ. Clinical observation of ambroterol oral liquid in treatment of mycoplasma pneumonia in children. Health friend. 2022(22):258-60.
69. LI XM. Clinical analysis of ambroterol oral liquid in treatment of bronchitis in children. China Health Industry. 2012;9(23):70.
70. LI YJ, HU QQ, ZHU YX, LI LX. Clinical efficacy of methylprednisolone combined with Yi Tan Jing in the treatment of refractory pneumonia in children. Practical Pharmacy and Clinical Remedies. 2018;21(12):1394-7.
71. LI YX, LI GC, XIE H, QIAN YM. The comparison of the therapeutic effect of yitanjing and ambroxol on 76 cases of bronchopneumonia in children. Medical Innovation of China. 2009;6(22):40-1.
72. LI ZY. Clinical efficacy of ambrocol on asthmatic disease in children. Clinical Medicine. 2014;34(06):28-9.
73. LIANG JB. Observation on curative effect of ambroterol oral liquid in treatment of bronchiolitis. China Health Care & Nutrition. 2012(5):261.
74. LIANG XH. Curative effect of ambroterol oral liquid combined with azithromycin on mycoplasma pneumonia in children. Journal of Qiqihar University of Medicine. 2017;38(24):2909-10.
75. LIANG ZF, NING HW. Clinical efficacy and safety of ambroterol oral liquid combined with pidotimod oral liquid in the treatment of children with mite allergic asthma. Chinese Journal of Biochemical and Pharmaceuticals. 2017;37(4).
76. LIN JR. Clinical effect of ambroterol oral solution in the treatment of children with bronchial pneumonia. Chinese Journal of Medical Device. 2019;32(20):113-4.
77. LIN XY. Evaluation of clinical therapeutic effect of Ambroxol Hydrochloride and Clenbuterol Hydrochloride Oral solution in treating children-Pneumonia. Yiyao Qianyan. 2015(25):15-,6.
78. LIN Y. Efficacy and safety of Bolicaniol and ambroterol combined with Montelukast in the treatment of infants with wheezing. China Health Care & Nutrition. 2018;28(19):270.
79. LIN YM, LI YP. Clinical effect of ambroxol oral liquid in the treatment of infantile bronchopneumonia. Clinical Research and Practice. 2019;4(26):98-9.
80. LING C. Curative effect of ambroterol oral liquid combined with azithromycin in the treatment of mycoplasma pneumonia in children. Internal Medicine of China. 2014;9(01):38-9.
81. LIU HP, BAO YX, Tian Y, CAO LF, DONG W, NI SH, et al. A multicenter clinical study on the treatment of infantile wheezing with combined descending steps. Proceedings of the 2017 Shanghai Medical Association Allergy Annual Conference; 上海 2017. p. 76-.
82. LIU L, WANG DJ. Clinical efficacy and effect of ambroterol oral liquid on pulmonary function in children with bronchiolitis. Chinese Remedies & Clinics. 2018;18(08):1365-7.
83. LIU ML, ZHANG G. Observation of Effect of Ambroxol Hydrochloride and Clenbuterol Hydrochloride Oral Solution inTreatment of Bronchopneumonia. Chinese Journal of Misdiagnostics. 2009;9(36):8843-4.

84. LIU XH. Therapeutic effect of ambroterol oral liquid on 36 cases of acute bronchitis in children. Medical Aesthetics and Cosmetology. 2015(5):751-.
85. LIU XJ. Clinical observation of ambroterol oral solution in treatment of acute bronchiolitis in children. China Medicine. 2011;06(10):1229-30.
86. LIU XJ. The efficacy inquiry of ambroxol and clenbuterol oral solution in pediatric bronchitis treatment. Clinical Research and Practice. 2017;2(01):74-5.
87. LIU XL. Therapeutic effect of ambroterol oral liquid on 90 cases of bronchiolitis. The Journal of Medical Theory and Practice. 2012;25(23):2950+85.
88. LIU XL. Curative effect of 25% magnesium sulfate injection and ambroterol oral liquid in the treatment of bronchiolitis. Guide of China Medicine. 2012;10(18):95-6.
89. LIU YT, JING XW. The clinical observation of Ambroxol Hydrochloride and Clenbuterol Hydrochloride Oral Solution on the treatment of infantile bronchitis. Chinese Journal of Clinical Rational Drug Use. 2012;5(11):49-50.
90. LONG P, YI RS, LAING ZX, LI X, WEI B. Therapeutic effect of ambroterol oral liquid in adjuvant treatment of 50 cases of infant pneumonia. Hainan Medical Journal. 2008;19(3):111-2.
91. LU JS. Observation of Children with Asthmatic Disease Treatment Effect of Am-brocol Oral Solution. Modern Diagnosis & Treatment. 2017;28(05):845-7.
92. LU M, TAN JF. Analysis of ambrocol in treating infant bronchiolitis. Journal of Clinical Pediatrics. 2010;28(05):489-90.
93. LUO HZ, ZHOU CL. Curative effect of ambroterol oral liquid combined with azithromycin in the treatment of mycoplasma pneumonia in children. Chinese Community Doctors. 2013;15(08):111.
94. LV XP. Therapeutic effect of ambroterol oral liquid on 93 cases of acute lower respiratory tract infection in children. Chinese Community Doctors. 2012;14(09):153.
95. MA JG. Clinical observation of ambroterol oral solution for adjuvant treatment of bronchiolitis in children. China Medical Engineering. 2016;24(03):51-2.
96. MA LP. Clinical observation of Yitanjing oral liquid in adjuvant treatment of children with bronchiolitis. Chinese Community Doctors. 2012;14(26):122.
97. MA LW. Observation on efficacy of ambroxol hydrochloride and clenbuterol hydrochloride on bronchiolitis. Journal of Pediatric Pharmacy 2007(03):46-7.
98. MA YY. Observation of curative effect of ambroterol oral liquid on bronchopneumonia. Chinese Community Doctors. 2013;15(04):103.
99. MAO Y, CHEN T. Therapeutic effect of ambroterol oral liquid in the treatment of bronchiolitis. China Foreign Medical Treatment. 2011;30(27):121-2.
100. MEI X, WANG HF, LI K. Observation of curative effect of Yi Tanjing in the adjuvant treatment of 112 cases of lower respiratory tract infection in children. World Health Digest 2009;6(28):139-40.
101. MENG LZ, ZHAO SX, LI XY, ZHAO Y, WANG HL. Clinical analysis of ambroterol oral liquid for adjuvant treatment of bronchophenitis in infants. Chinese Remedies & Clinics. 2009;9(08):759-60.
102. MENG XH. Clinical effect of ambroterol on 280 children with bronchiolitis. Guide of China Medicine. 2012;10(25):159-60.
103. MIAO P. Application of ambroterol oral liquid in the treatment of children with bronchiolitis. China Practical Medicine. 2014;9(14):168-9.
104. MO CX. Effect analysis of ambroxol hydrochloride and clenbuterol hydrochloride oral solution in the adjunctive treatment of mycoplasma pneumonia in children. China Modern Medicine. 2013;20(08):90-1.
105. PENG JB, CHEN BB, DENG CH. Clinical study of 44 cases of severe pneumonia treated with ambroterol. Heilongjiang Medicine Journal 2015;28(02):331-2.
106. PENG KY, ZHAO RL. Clinical effect of ambroterol oral liquid on bronchopneumonia in children. Good Health for All. 2014;8(13):139-40.
107. PENG WJ, FAN J, CHEN CH. The clinical observation of the treatment of mycoplasma pneumoniae pneumonia with Jinzhen Oral Liquid and clarithromycin. Journal of Pediatrics of Traditional Chinese Medicine. 2012;8(01):15-8.
108. PENG XH, WANG LH. Analysis of ambroxol hydrochloride and clenbuterol hydrochloride oral solution in treating mycoplasma pneumonia. China Medicine. 2011;06(8):981-2.
109. QIU LJ, LI F, Wang J, BI MR, ZHU WW. Observation on the curative effect of ambroterol oral liquid in the treatment of bronchopneumonia in infants. Frontiers in Pharmaceutical Sciences. 2013;16(09):1381-3.
110. QU JN. Efficacy of Montelukast and ambroterol in improving respiratory symptoms caused by mycoplasma pneumoniae infection in children. Medicine and Human. 2014;27(10).
111. QU X. Observation on the effect of yitanzin combined with methylprednisolone in treatment of refractory pneumonia in children. The Journal of Medical Theory and Practice. 2020;33(06):964-5.
112. SAHO S. Analysis of curative effect of ambroterol oral liquid on mycoplasma pneumoniae pneumonia in children. Chinese Journal of Modern Drug Application. 2016;10(04):140-1.

113. SHAN LC, HOU P, WANG ZJ, CHEN N, SHANG YX. Clinical observation on symptomatic treatment of acute bronchopneumonia with ambroterol oral liquid. Chinese Journal of Practical Pediatrics. 2011;26(04):306-8.
114. SHE NH. Analysis of therapeutic effect of ambroterol oral liquid on bronchopneumonia in infants. Chinese Journal of Women and Children Health. 2013;4(02):48.
115. SHEN SS. Clinical observation of ambroterol oral solution for adjuvant treatment of bronchiolitis in children. Journal of North Pharmacy. 2014;11(05):54.
116. SHEN WY, DAI WY. Clinical analysis of ambroterol oral liquid combined with azithromycin in the treatment of mycoplasma pneumonia in children. Chinese Journal of Primary Medicine and Pharmacy. 2012;19(7):1083-4.
117. SHI SM, LONG P, YI RS, LIANG ZX. Observation on the curative effect of Yitanjing in the treatment of infants with asthma and pulmonary infection. Hainan Medical Journal. 2011;22(21):75-6.
118. SHU Y. Observation on the Effect of Ambroterol Oral Solution in the Treatment of Bronchitis in Children. Journal of Chinese and Foreign Medicine and Pharmacy Research. 2023;2(27).
119. SONG BH. Observation on the curative effect of ambroteroxor hydrochloride on lower respiratory tract infection in children. Chinese Community Doctors. 2012;14(26):143-.
120. SONG P. Clinical experience of ambroterol oral liquid in the treatment of bronchiolitis. Chinese Medical Abstracts (Pediatrics). 2007(03):200-1.
121. SONG YY, ZHU B. Application of Yitanjing oral liquid in children with pneumonia. Proceeding of Clinical Medicine. 2009;18(7):548-9.
122. SUN Y. Therapeutic effect of ambroterol oral liquid on 1300 cases of bronchopneumonia in infants. World Latest Medicine Information. 2015;15(98):82-3.
123. TAN QY, LIN C. Effect of Abrutro -Oral Liquid for the Clinical Treatment of Pediatric Bronchitis and Its Influence on Serum C-reactive Protein (CRP) and Tu-mor Necrosis Factor α (TNF-α) in Children. Systems Medicine. 2017;2(20):78-80.
124. TAN YL. Clinical effect of ambroterol oral liquid on children with bronchitis. Good Health for All. 2016;10(5):153-.
125. TANG LF. Clinical efficacy of ambroterol in the adjuvant treatment of infant asthmatic bronchitis. Shanxi Medical Journal. 2009;38(07):642-3.
126. TANG Q. Effect analysis of ambroterol oral liquid in the treatment of children with bronchiolitis. Journal of North Pharmacy. 2012;9(07):105.
127. TANG Y. Clinical observation of ambroterol oral liquid in the treatment of pneumonia in children. Chinese Journal of Misdiagnostics. 2008(07):1559-60.
128. TIAN T. Evaluation of ambroterol oral liquid in clinical treatment of bronchitis in children. Health Guide. 2018(8):75.
129. TIAN YH. Clinical observation of 96 cases of bronchopneumonia in children treated by Yi Tanjing. China Medical Engineering. 2012;20(07):122.
130. TONG FM. Comparative study on the efficacy of two methods in treating acute bronchitis in children. China Medical Engineering. 2014;22(03):131.
131. W.T YMJD, S MRGA. Clinical observation of 100 children with pneumonia treated with Yi Tanjing. Medical Innovation of China. 2012;9(20):127.
132. WANG AS, DU QL. Observation of curative effect of ambroterol oral liquid on respiratory symptoms of children with bronchopneumonia. Chinese Community Doctors. 2007(17):84.
133. WANG AY, DING XY. The clinical observation of oral ambroxol clenbuterol on the treatment of capillary bronchitis. China Modern Medicine. 2011;18(04):37-8.
134. WANG D. Effect analysis of ambroterol oral liquid for clinical treatment of bronchitis in children. China Health Care & Nutrition. 2021;31(30):216.
135. WANG DY, SHI LH, WANG Y. Effect of ambroterol oral liquid on pulmonary function in children with bronchiolitis. World Health Digest 2013(6):177-8.
136. WANG F, WANG S, LI YP. Therapeutic effect of ambroterol oral liquid on 60 cases of bronchiolitis in children. Clinical Research and Practice. 2008(04):156.
137. WANG G. Feasibility analysis of Yi Tanjing combined with routine treatment of bronchopneumonia in children. Capital Medicine. 2018;25(14):54-5.
138. WANG K, D, XU PF. Therapeutic effect of ambroterol oral liquid in adjuvant treatment of mycoplasma pneumonia in children. Chinese Journal of Pharmacoepidemiology. 2013;22(07):347-9.
139. Wang L. Curative Efficacy of Ambrocol for Lower Respiratory Tract Infection in Children. Chinese Journal of General Practice. 2007(07):609-10.
140. WANG LF, HUANG XM, SI YH. Observation on the curative effect of ambroterol oral liquid in the treatment of lower respiratory tract infection in children. Guide of China Medicine. 2010;8(06):104-5.

141. WANG MM, XING WM, Zhang L, HAN XM. Clinical research of Qingfei Oral Liqulid and Xiyanping Injection in treatment of children with human bocavirus pneumonia. Chinese Traditional and Herbal Drugs. 2017;48(18):3802-6.
142. WANG MR, SHI H, QIAN L. Therapeutic effect of ambroxol oral solution on children with asthmatic bronchial pneumonia. China Modern Doctor. 2018;56(24):57-9.
143. WANG MZ. Clinical analysis of ambroterol oral liquid in treatment of bronchitis in children. China Practical Medicine. 2015(23):191-2.
144. WANG PL, WANG CM. Clinical efficacy of ambroterol oral solution combined with Montelukast in the treatment of bronchiolitis. World Latest Medicine Information. 2021;21(46):168-9.
145. WANG Q. Clinical observation of children bronchopneumonia treated with ambroterol oral liquid. Chinese Journal of Ethnomedicine and Ethnopharmacy. 2013;22(12):87-8.
146. WANG Q, SUN LJ, JIANG XH. Ambroterol oral liquid for the treatment of 30 cases of bronchopneumonia in children. Clinical Journal of Medical Officers 2006(05):589+99.
147. WANG SH. Observation of curative effect of ambroterozox on bronchiolitis. Journal of Chinese and Foreign Medicine and Pharmacy Research. 2010;8(24):56-7.
148. WANG Y, LIN YP, SU HL, LIN X. Clinical effect of ambroterol on 98 children with bronchiolitis. Guide of China Medicine. 2013;11(23):102-3.
149. WANG Y, SHI LH, ZHANG Q. Effect of ambroterol oral liquid on pulmonary function in children with bronchiolitis. China Health Monthly. 2011;30(10):233-4.
150. WANG ZK, WU BZ, WU JY. Curative effect of ambroterol oral liquid on 98 cases of bronchiolitis. Guangxi Medical Journal. 2009;31(05):714-5.
151. WEI B, LI QY, JIANG J, LI W. Curative effect of ambroxol hydrochloride and clenbuterol hydrochloride oral solution in mycoplasma pneumonia children. China Medicine. 2011;06(8):978-80.
152. WEI CH, CHEN HN. Effect of Ambroxol and Clenbuterol Oral Solution in the treatment of children with bronchitis. China Medical Herald. 2015;12(03):114-7+26.
153. WEI D, ZHANG XL, ZHAO XL. Efficacy of Ambroxol Castro for the Treatment of Children with Bronchopneumonia and the Impact on IL-6 and CRP. Journal of Clinical Research. 2013(8):1589-90.
154. WEI H, PENG L. Therapeutic effect of ambroterol oral liquid in the adjuvant treatment of children with asthmatic diseases. Central China Medical Journal. 2008(04):277-8.
155. WEI HJ, GUO H, YU HX. Observation of curative effect of Yi Tanjing adjuvant on 64 cases of bronchopneumonia in children. Medical Information. 2011;24(09):4382.
156. WEN H, LIU XL. Therapeutic effect of ambroterol oral liquid on 200 cases of bronchopneumonia in infants. Chinese Community Doctors. 2013;15(21):52-3.
157. WU QF. Clinical observation of ambroterol in treatment of acute bronchitis in children. Frontiers in Pharmaceutical Sciences. 2012;15(10):1463-4.
158. XIA H. Observation of curative effect of ambroterozox in children with lower respiratory tract infection. Asia-Pacific Traditional Medicine. 2010;6(6):72-3.
159. XIA HJ, TIAN QY, ZHANG WY, LI XX. Observation on the clinical effect of ambroterol oral liquid on bronchophenitis in children. Chinese Journal of Modern Drug Application. 2013;7(04):52-3.
160. XIA JX, LV Q. Ambroterol combined with budesonide aerosol inhalation in the treatment of acute asthma attack in infants. Frontiers in Pharmaceutical Sciences. 2015;18(03):427-9.
161. XIAO WH, PENG XH. Clinical study of Bolicanyl and ambroterol combined with Montelukast in the treatment of pediatric asthma. Contemporary Medicine. 2014;20(23):142-3.
162. XIAO YH. Clinical effect of pediatric Feirekechuan oral liquid on mycoplasma pneumoniae bronchitis. The Medical Forum. 2018;22(32):4527-8.
163. XIE TT, LU F, LAING F, TIAN HX, ZUO YY. Intervention effect of pidotimod combined with ambroxol on the serum levels of KL-6,GM-CSF,inflammatory factors and immune function in children with Mycoplasma pneumonia. Laboratory Medicine and Clinic. 2022;19(08):1040-4.
164. XIE YL. Clinical Effect Evaluation of Children with Bronchiolitis in Application of Ambrocol Oral Solution Combined with Compound Ipratropium Bromide Oxygen Inhalation Therapy. Medical Innovation of China. 2015;12(25):41-3.
165. XU HX, HOU HY. Curative effect of ambroterol oral liquid in the treatment of 100 cases of bronchiolitis. National Medical Frontiers of China. 2007(06):125-6.
166. XU JF, XIAO YF, LI RH, MA Y. Effect of ambroterol oral solution combined with azithromycin dry suspension in adjuvant treatment of mycoplasma pneumonia in children. Care & Health. 2022(6):13-5.
167. XU SS, ZhANG L, ZHAO EY, DONG LL, REN Q. Clinical Observation of 96 Cases of Acute Asthmatic Bronchitis Treated by Xiaoer Dingchuan Oral Liquid. Asia-Pacific Traditional Medicine. 2021;17(07):90-2.
168. XU XJ. Analysis of curative effect of ambroterol oral solution on acute respiratory tract infection in children. Electronic Journal of Clinical Medical Literature. 2018;5(47):153+66.

169. XU XQ, ZHOU JG, DI W, HE RR. Observation on curative effect of ambroterol oral liquid in treatment of bronchiolitis. Chinese Journal of Western and Chinese Medicine. 2007;5(5).
170. XU YF. Therapeutic effect of ambroterol oral solution in the adjuvant treatment of mycoplasma pneumoniae pneumonia in children. China Practical Medicine. 2014;9(23):132-3.
171. XU YP, LU P. Clinical observation of ambroterol in adjuvant treatment of lower respiratory tract infection in children. Guide of China Medicine. 2009;7(05):68.
172. XUE P. Observation on the curative effect of Yitanjing in the treatment of infant asthmatic diseases. Practical Pharmacy and Clinical Remedies. 2006(03):157-8.
173. XUE YY. Analysis of therapeutic effect of aminoterol oral liquid on respiratory symptoms in children with bronchiolitis. Chinese Journal of Practical Medicine. 2008;35(5):89.
174. YAN L. Therapeutic effect of ambroterol on children with bronchiolitis. Contemporary Medicine. 2015;21(04):127-8.
175. YAN XM. The efficacy of antibiotics combined with ambroxolhydrochlorideand clenbuterol hydrochloride oral solution in the treatment ofpediatric pneumonia. Love and Health. 2024;30(05):58-60.
176. YANG J, SUN HJ, WANG Y. Observation on the effect of montelukast and ambroxol hydrochloride in treatment respiratory tract symptoms in children with mycoplasma pneumoniae infection. Chinese Journal of Primary Medicine and Pharmacy. 2013;20(14):2095-6.
177. YANG JL. Therapeutic effect of ambroterol oral liquid in the treatment of bronchitis. Shanxi Medical Journal. 2014;43(09):1041-2.
178. YANG XQ, LI SG, LIANG JL. Clinical effect of bricanyl and ambroxol and clenbuterol in respective combination with montelukast in the treatment of infant wheezing. China Practical Medical 2015;10(10):17-8.
179. YANG YZ. Observation on the curative effect of interferon and itanidine in the treatment of infantile bronchiolitis. Chinese Journal of Clinical Rational Drug Use. 2012;5(03):44.
180. YANG ZM. Experience in the clinical treatment of 87 cases of infantile pneumonia treated with ambroteroxib. Chinese Journal of Ethnomedicine and Ethnopharmacy. 2014;23(09):90.
181. YANG ZM, FAN MY. Application of ambroterol oral liquid in children with bronchopneumonia. The Medical Forum. 2012;16(35):4636-7.
182. YANG ZM, FAN MY. Therapeutic effect of ambroterol oral liquid on bronchopneumonia in children. The Medical Forum. 2012;16(11):1379-80.
183. YE ZQ. Comparison of curative effect of Xuanfei Zhisou mixture and ambroterol oral liquid in the treatment of acute bronchitis in children. Journal of Clinical Pulmonary Medicine. 2015;20(7).
184. YIN BJ, WANG MM, HU ZY. Therapeutic effect of ambroterol oral liquid combined with atomized pulmicresol and Boliconil on 80 cases of bronchial asthma in children. Chinese Community Doctors. 2012;14(30):20.
185. YU XM. Curative effect of ambroterol on bronchiolitis. Frontiers in Pharmaceutical Sciences. 2011;14(11):1655-6.
186. YU YJ. Efficacy and safety of ambroxol combined with montelukast for children with bronchiolitis. Sichuan Journal of Physiological Sciences. 2020;42(01):71-4.
187. YUAN QL. Evaluation of therapeutic effect of ambroterol oral liquid in the treatment of bronchitis in children. Journal of North Pharmacy. 2017;14(11):88-9.
188. YUAN SF. Curative effect of ambroterol on bronchiolitis. Zhejiang Clinical Medical Journal. 2007;9(12):1631.
189. YUAN Y, JU LN. Observation on the curative effect of ambroterol oral liquid in the treatment of bronchopneumonia in infants. Medical Innovation of China. 2011;8(11):52-4.
190. ZHAI YH, ZHU TJ. Observation on the curative effect of Yitanjing in the treatment of children with asthmatic bronchitis. ournal of Yangtze University(Natural Science Edition). 2012;9(10):25-6.
191. ZHAN XM, YE JF, ZOU XH. Curative efficacy of Ambrocol in the treatment of children with pneumonia. Strait Pharmaceutical Journal. 2009;21(04):109-10.
192. ZHANG CY, ZHANG LP, FAN L. Therapeutic effect of ambroterol oral liquid in the treatment of infant bronchiolitis. Medical Innovation of China. 2011;8(10):75-6.
193. ZHANG GL, ZHU M. The observation on treatment of infant bronchopneumonia with ambrocol. Journal of Pediatric Pharmacy 2005(04):44-5.
194. ZHANG GM. Comparative study on the effect of Feilike mixture and ambroterol oral liquid on acute bronchitis in children. Clinical Journal of Chinese Medicine. 2018;10(32).
195. ZHANG H. Application of ambroterol in the treatment of bronchopneumonia. China Health Standard Management. 2015;6(23):122-3.
196. ZHANG HM. Treatment of 86 children with mycoplasma pneumonia by azithromycin combined with ambroterol oral solution. Good Health for All. 2013;7(16):16.

197. ZHANG HY, DENG YL. Observation of curative effect of ambroterozox in children with lower respiratory tract infection. Journal of Modern Medicine & Health. 2009;25(18):2792-3.
198. ZhANG L, CEHN J. Therapeutic effect of ambroterozox on 337 cases of bronchiolitis. Laboratory Medicine and Clinic. 2012;9(22):2835-6.
199. ZHANG LM. Evaluation of therapeutic effect of ambroterol oral liquid in the treatment of bronchitis in children. Health Required. 2019(7):74-5.
200. ZHANG LZ. Observation of the effect of ambroxol and clenbuterol oral solution in the treatment of children with pneumonia. Journal of Clinical Pulmonary Medicine. 2007(02):124-5.
201. ZHANG M, L. Clinical efficacy of ambroterol combined with azithromycin in children with mycoplasma pneumonia. Practical Clinical Journal of Integrated Traditional Chinese and Western Medicine. 2018;18(05):34-6.
202. ZHANG M. Analysis of therapeutic effect of ambroterol oral liquid on bronchopneumonia in infants. China Health Care & Nutrition. 2019;29(16):317.
203. ZHANG Y, JIN R, LU G, HUANG L, ZHANG D. Observation on the curative effect of interferon nebulization inhalation and yitanjing in the treatment of 30 cases of bronchiolitis. Guizhou Medical Journal. 2011;35(09):808-10.
204. ZHANG YJ. Clinical study of Jinzhen oral liquid combined with clarithromycin in treatment of mycoplasma pneumoniae pneumonia in children [硕士]2008.
205. ZHANG ZH. Clinical effect of ambroterol on 60 cases of community-acquired pneumonia. Chinese Journal for Clinicians. 2013;41(10):59-60.
206. ZHAO HR, SU CY. Clinical study on ambroxol and clenbuterol combined with salbutamol in treatment of bronchial asthma in children. Drugs & Clinic. 2019;34(04):1034-7.
207. ZHAO J. Clinical effect of ambroxol oral solution in the treatment of children with bronchial pneumonia. Clinical Research and Practice. 2018;3(30):69-70.
208. ZHAO L. Curative effect of ambroterol combined with azithromycin on 60 cases of mycoplasma pneumonia in children. Maternal and Child Health Care of China. 2013;28(26):4382-3.
209. ZHAO XY. Therapeutic effect of ambroterol oral solution on children with bronchiolitis. Electronic Journal of Clinical Medical Literature. 2016;3(12):2423-4.
210. ZHAO YQ. Analysis of curative effect of ambroterol on children with bronchiolitis. China Medical Equipment. 2014;11(S2):421.
211. ZHAO YZ. Evaluation on the effect of salbutamol combined with ambroxol in the treatment of children with bronchial asthma. Chinese Journal of Modern Drug Application. 2021;15(17):163-5.
212. ZHAO ZQ, FU YQ, ZHU LL, KUANG QG, WEN YH. Effect comparison of ammonium bromide and ambroxol hydrochloride in the treatment of bronchopneumonia in children. China Modern Medicine. 2015;22(06):119-20+23.
213. ZHEN DX, WEN H. Observation on the curative effect of Yitanjing in the treatment of asthmatic pneumonia. Journal of Modern Medicine & Health. 2007(15):2292.
214. ZHOU J. Clinical analysis of 1000 cases of bronchopneumonia in children. Chinese Community Doctors. 2012;14(21):136.
215. ZHOU JY. Curative effect of oral ambroterol in the treatment of bronchiolitis. Medical Information. 2012;25(2):514-.
216. ZHOU LG. Clinical observation on symptomatic treatment of acute bronchopneumonia with ambroterol oral liquid. Good Health for All. 2016;10(05):180-1.
217. ZHOU Q. Influence of Ambroxol Hydrochloride and Clenbuterol Hydrochloride Oral Solution on airway function of children with capillary bronchitis. Contemporary Medicine. 2013;19(13):144-5.
218. ZHOU X. Effect of Interferon and Yitanjing on infants with bronchiolitis: 40 cases observation. Journal of Hainan Medical University. 2009;15(05):460-1+4.
219. ZHOU XC, MENG XJ. Clinical observation of ambroterol oral solution (Itanidine) in the treatment of children with asthmatic pneumonia. China Health Care & Nutrition. 2013(9):5296-.
220. ZHOU XL, ZHU FS. Clinical analysis of 100 cases of pediatric bronchiolitis treated with ambroterol hydrochloride oral liquid. Journal of Clinical and Experimental Medicine. 2008(05):158.
221. ZHOU ZW, HE B. Effect of ambroterol plus compound ipratropium bromide in the treatment of children with bronchiolitis. Diet Health. 2018;5(32):59-60.
222. ZHU SL. Effect of ambroterol oral liquid on pulmonary function and inflammatory factor levels in children with bronchial pneumonia. Modern Medicine and Health Research. 2021;5(02):60-2.
223. ZHU SY. Effect of Ambroxol Hydrochloride and Clenbuterol Hydrochloride Oral Solution in Treatment of Bronchopneumonia in 72 Infants. Chinese Journal of Applied Clinical Pediatrics. 2012;27(4):308-9.
224. ZHU XY, YE MY, FANG F. Clinical analysis of yitanjing oral assisted treatment of bronchopneumonia in infants. Chinese Journal of Aesthetic Medicine. 2010;19(z4):206.

225. ZHU YE. Clinical effect of ambroterol in the adjuvant treatment of mycoplasma pneumonia in children. Practical Clinical Journal of Integrated Traditional Chinese and Western Medicine. 2021;21(02):89-91.
226. ZHU YT. Clinical observation of ambroterol oral liquid in treating 96 cases of bronchophenitis in infants. China Health Care & Nutrition. 2015;25(15):251.
227. ZHUO QC. Clinical efficacy of ambroterol oral liquid in improving respiratory symptoms in children with bronchitis. Guide of China Medicine. 2013;11(30):23-4.

Supplementary 3 Evidence mapping results

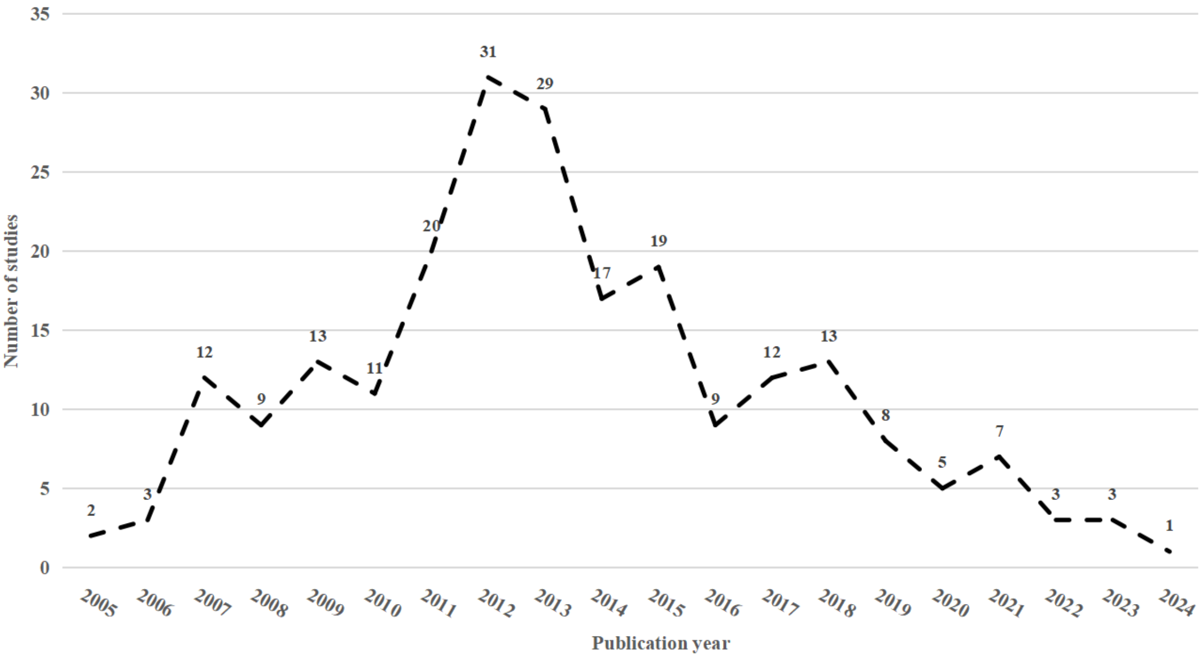

Figure S1 Trends in the publication years of randomized controlled trials on ambroxol hydrochloride and clenbuterol hydrochloride oral solution

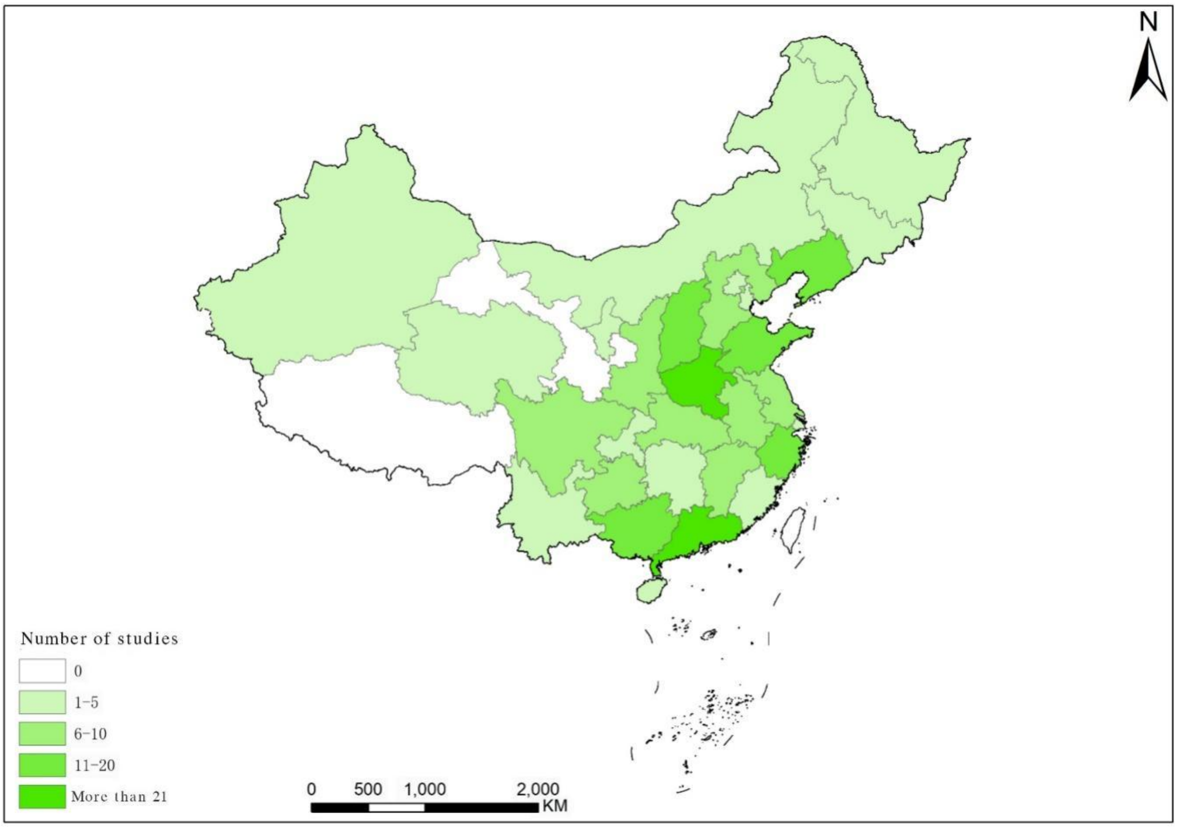

Figure S2 Geographical distribution of randomized controlled trials on ambroxol hydrochloride and clenbuterol hydrochloride oral solution

Table S1 Sample size distribution characteristics of randomized controlled trials on ambroxol hydrochloride and clenbuterol hydrochloride oral solution

Sample size range	Actual sample size range	Number of studies	Percentage
31-60	32-60	17	7.49%
61-100	61-100	123	54.19%
101-200	110-200	75	33.04%
Over 200	206-1300	12	5.29%

Table S2 Summary of intervention comparisons in randomized controlled trials on ambroxol hydrochloride and clenbuterol hydrochloride oral solution

Intervention vs. Control	Number of studies	Percentage
AHCHOS + Conventional treatment vs. Conventional treatment	98	43.17%
AHCHOS + Conventional treatment vs. Ambroxol + Conventional treatment	19	8.37%
AHCHOS vs. Ambroxol	12	5.29%
AHCHOS + Conventional treatment vs. antitussive + Conventional treatment	10	4.41%
AHCHOS + Antibiotic + Conventional treatment vs. Antibiotic + Conventional treatment	10	4.41%
AHCHOS + Antibiotic vs. Antibiotic	9	3.96%
AHCHOS vs. antitussive	8	3.52%
AHCHOS + Compound Ipratropium Bromide Aerosol + Conventional treatment vs. Conventional treatment	4	1.76%
AHCHOS + interferon + Conventional treatment vs. Conventional treatment	4	1.76%
AHCHOS + Montelukast + Conventional treatment vs. Conventional treatment	4	1.76%
AHCHOS vs. Conventional treatment	3	1.32%
AHCHOS vs. antitussive + Conventional treatment	3	1.32%
AHCHOS + Antibiotic vs. Antibiotic + Ambroxol	3	1.32%
AHCHOS + Montelukast vs. bricanyl + Montelukast	3	1.32%
AHCHOS + Montelukast + Conventional treatment vs. Montelukast + Conventional treatment	3	1.32%

AHCHOS + budesonide + Conventional treatment vs. budesonide + Conventional treatment	2	0.88%
AHCHOS + Conventional treatment vs. Salbutamol Sulfate + Mucosolvan + Conventional treatment	2	0.88%
AHCHOS + Antibiotic vs. Antibiotic + antitussive	2	0.88%
AHCHOS vs. Compound Ammonium Chloride Oral Solution	1	0.44%
AHCHOS + Conventional treatment vs. Ambroxol + Salbutamol Sulfate Tablets + Conventional treatment	1	0.44%
AHCHOS + Conventional treatment vs. Ambroxol + Terbutaline Sulphate + Conventional treatment	1	0.44%
AHCHOS + Conventional treatment vs. budesonide\Asarone + Conventional treatment	1	0.44%
AHCHOS + Conventional treatment vs. Pidotimod + Conventional treatment	1	0.44%
AHCHOS + Conventional treatment vs. Xiaoer Dingchuan oral liquid + Basic treatment	1	0.44%
AHCHOS + Conventional treatment vs. General cough and expectorant drugs + Conventional treatment	1	0.44%
AHCHOS + Conventional treatment vs. guaiacolsulfonicacidpotassium + Conventional treatment	1	0.44%
AHCHOS + Compound Ipratropium Bromide Aerosol + Conventional treatment vs. Ambroxol + Sodium Succinate Injection + Antibiotic + Conventional treatment	1	0.44%
AHCHOS + Montelukast vs. Montelukast	1	0.44%
AHCHOS Ambroxol Hydrochloride and Clenbuterol Hydrochloride Oral Solution		

Table S3 Risk of bias of 227 randomised controlled trials included in evidence mapping

Study ID	Random sequence generation (selection bias)	Allocation concealment (selection bias)	Blinding of participants and personnel (performance bias)	Blinding of outcome assessment (detection bias)	Incomplete outcome data (attrition bias)	Selective reporting (reporting bias)	Other bias	Overall
Shen 2012	Low risk	Unclear risk	Unclear risk	Unclear risk	Low risk	Low risk	Low risk	Unclear risk
Zhai 2012	Unclear risk	Unclear risk	Unclear risk	Unclear risk	Low risk	Low risk	Low risk	Unclear risk
Peng 2012	Unclear risk	Unclear risk	Unclear risk	Unclear risk	Low risk	Low risk	Low risk	Unclear risk
Lv 2012	Unclear risk	Unclear risk	Unclear risk	Unclear risk	Low risk	Low risk	Low risk	Unclear risk
Li 2012	Unclear risk	Unclear risk	Unclear risk	Unclear risk	Low risk	Low risk	Low risk	Unclear risk
Ding 2012	Unclear risk	Unclear risk	Unclear risk	Unclear risk	Low risk	Low risk	Low risk	Unclear risk
Zhang 2011	Unclear risk	Unclear risk	Unclear risk	Unclear risk	Low risk	Low risk	Low risk	Unclear risk
Yuan 2011	Unclear risk	Unclear risk	Unclear risk	Unclear risk	Low risk	Low risk	Low risk	Unclear risk
Wei 2011a	Unclear risk	Unclear risk	Unclear risk	Unclear risk	Low risk	Low risk	Low risk	Unclear risk
Wei 2011b	Unclear risk	Unclear risk	Unclear risk	Unclear risk	Low risk	Low risk	Low risk	Unclear risk
Wang 2011	Unclear risk	Unclear risk	Unclear risk	Unclear risk	Low risk	Low risk	Low risk	Unclear risk
Shi 2011	Unclear risk	Unclear risk	Unclear risk	Unclear risk	Low risk	Low risk	Low risk	Unclear risk
Huo 2011	Unclear risk	Unclear risk	Unclear risk	Unclear risk	Low risk	Low risk	Low risk	Unclear risk
He 2011	Unclear risk	Unclear risk	Unclear risk	Unclear risk	Low risk	Low risk	Low risk	Unclear risk
Fan 2011	Low risk	Unclear risk	Unclear risk	Unclear risk	Low risk	Low risk	Low risk	Unclear risk
Deng 2011	Unclear risk	Unclear risk	Unclear risk	Unclear risk	Low risk	Low risk	Low risk	Unclear risk

Shan 2011	Unclear risk	Unclear risk	Unclear risk	Unclear risk	Low risk	Low risk	Low risk	Unclear risk
Zhu 2010	Unclear risk	Unclear risk	Unclear risk	Unclear risk	Low risk	Low risk	Low risk	Unclear risk
Xia 2010	Low risk	Unclear risk	Unclear risk	Unclear risk	Low risk	Low risk	Low risk	Unclear risk
Bai 2010	Unclear risk	Unclear risk	Unclear risk	Unclear risk	Low risk	Low risk	Low risk	Unclear risk
Zhan 2009	Unclear risk	Unclear risk	Unclear risk	Unclear risk	Low risk	Low risk	Low risk	Unclear risk
Xu 2009	Unclear risk	Unclear risk	Unclear risk	Unclear risk	Low risk	Low risk	Low risk	Unclear risk
Tang 2009	Unclear risk	Unclear risk	Unclear risk	Unclear risk	Low risk	Low risk	Low risk	Unclear risk
Song 2009	Unclear risk	Unclear risk	Unclear risk	Unclear risk	Low risk	Low risk	Low risk	Unclear risk
Meng 2009	Unclear risk	Unclear risk	Unclear risk	Unclear risk	Low risk	Low risk	Low risk	Unclear risk
Liu 2009	Unclear risk	Unclear risk	Unclear risk	Unclear risk	Low risk	Low risk	Low risk	Unclear risk
Li 2009a	Unclear risk	Unclear risk	Unclear risk	Unclear risk	Low risk	Low risk	Low risk	Unclear risk
Li 2009b	Unclear risk	Unclear risk	Unclear risk	Unclear risk	Low risk	Low risk	Low risk	Unclear risk
Zhou 2008	Unclear risk	Unclear risk	Unclear risk	Unclear risk	Low risk	Low risk	Low risk	Unclear risk
Zhang 2008	Unclear risk	Unclear risk	Unclear risk	Unclear risk	Low risk	Low risk	Low risk	Unclear risk
Xue 2008	Unclear risk	Unclear risk	Unclear risk	Unclear risk	Low risk	Low risk	Low risk	Unclear risk
Wei 2008	Unclear risk	Unclear risk	Unclear risk	Unclear risk	Low risk	Low risk	Low risk	Unclear risk
Wang 2008	Unclear risk	Unclear risk	Unclear risk	Unclear risk	Low risk	Low risk	Low risk	Unclear risk
Tang 2008	Unclear risk	Unclear risk	Unclear risk	Unclear risk	Low risk	Low risk	Low risk	Unclear risk
Long 2008	Unclear risk	Unclear risk	Unclear risk	Unclear risk	Low risk	Low risk	Low risk	Unclear risk
Dong 2008	Unclear risk	Unclear risk	Unclear risk	Unclear risk	Low risk	Low risk	Low risk	Unclear risk
An 2008	Unclear risk	Unclear risk	Unclear risk	Unclear risk	Low risk	Low risk	Low risk	Unclear risk
Zhang 2007	Unclear risk	Unclear risk	Unclear risk	Unclear risk	Low risk	Low risk	Low risk	Unclear risk
Yuan 2007	Unclear risk	Unclear risk	Unclear risk	Unclear risk	Low risk	Low risk	Low risk	Unclear risk

Xu 2007a	Unclear risk	Unclear risk	Unclear risk	Unclear risk	Low risk	Low risk	Low risk	Unclear risk
Wang 2007a	Unclear risk	Unclear risk	Unclear risk	Unclear risk	Low risk	Low risk	Low risk	Unclear risk
Wang 2007b	Unclear risk	Unclear risk	Unclear risk	Unclear risk	Low risk	Low risk	Low risk	Unclear risk
Song 2007	Unclear risk	Unclear risk	Unclear risk	Unclear risk	Low risk	Low risk	Low risk	Unclear risk
Jiang 2007	Unclear risk	Unclear risk	Unclear risk	Unclear risk	Low risk	Low risk	Low risk	Unclear risk
He 2007	Unclear risk	Unclear risk	Unclear risk	Unclear risk	Low risk	Low risk	Low risk	Unclear risk
Guo 2007	Unclear risk	Unclear risk	Unclear risk	Unclear risk	Low risk	Low risk	Low risk	Unclear risk
Xue 2006	Unclear risk	Unclear risk	Unclear risk	Unclear risk	Low risk	Low risk	Low risk	Unclear risk
Wang 2006	Unclear risk	Unclear risk	Unclear risk	Unclear risk	Low risk	Low risk	Low risk	Unclear risk
Hong 2006	Low risk	Unclear risk	Unclear risk	Unclear risk	Low risk	Low risk	Low risk	Unclear risk
Zhang 2005	Unclear risk	Unclear risk	Unclear risk	Unclear risk	Low risk	Low risk	Low risk	Unclear risk
Huang 2005	Unclear risk	Unclear risk	Unclear risk	Unclear risk	Low risk	Low risk	Low risk	Unclear risk
Yan 2024	Unclear risk	Unclear risk	Unclear risk	Unclear risk	Low risk	Low risk	Low risk	Unclear risk
Shu 2023	Unclear risk	Unclear risk	Unclear risk	Unclear risk	Low risk	Low risk	Low risk	Unclear risk
Kang 2023	Low risk	Unclear risk	Unclear risk	Unclear risk	Low risk	Low risk	Low risk	Unclear risk
Cao 2023	Low risk	Unclear risk	Unclear risk	Unclear risk	Low risk	Low risk	Low risk	Unclear risk
Xu 2022	Unclear risk	Unclear risk	Unclear risk	Unclear risk	Low risk	Low risk	Low risk	Unclear risk
Xie 2022	Low risk	Unclear risk	Unclear risk	Unclear risk	Low risk	Low risk	Low risk	Unclear risk
Ou 2014	Low risk	Unclear risk	Unclear risk	Unclear risk	Low risk	Low risk	Low risk	Unclear risk
Yang 2013	Low risk	Unclear risk	Unclear risk	Unclear risk	Low risk	Low risk	Low risk	Unclear risk
Wang 2021	Low risk	Unclear risk	Unclear risk	Unclear risk	Low risk	Low risk	Low risk	Unclear risk
Li 2021	Low risk	Unclear risk	Unclear risk	Unclear risk	Low risk	Low risk	Low risk	Unclear risk
Lin 2019	Low risk	Unclear risk	Unclear risk	Unclear risk	Low risk	Low risk	Low risk	Unclear risk

Chen 2019	Unclear risk	Unclear risk	Unclear risk	Unclear risk	Low risk	Low risk	Low risk	Unclear risk
Zhang 2018a	Unclear risk	Unclear risk	Unclear risk	Unclear risk	Low risk	Low risk	Low risk	Unclear risk
Xiao 2018	Low risk	Unclear risk	Unclear risk	Unclear risk	Low risk	Low risk	Low risk	Unclear risk
Wang 2018a	Unclear risk	Unclear risk	Unclear risk	Unclear risk	Low risk	Low risk	Low risk	Unclear risk
Liu 2018	Low risk	Unclear risk	Unclear risk	Unclear risk	Low risk	Low risk	Low risk	Unclear risk
Li 2018	Low risk	Unclear risk	Unclear risk	Unclear risk	Low risk	Low risk	Low risk	Unclear risk
Wang 2017	Low risk	Unclear risk	Unclear risk	Unclear risk	Low risk	Low risk	Low risk	Unclear risk
Liu 2017a	Low risk	Unclear risk	Unclear risk	Unclear risk	Low risk	Low risk	Low risk	Unclear risk
Liang 2017a	Unclear risk	Unclear risk	Low risk	Unclear risk	Low risk	Low risk	Low risk	Unclear risk
Li 2017	Unclear risk	Unclear risk	Unclear risk	Unclear risk	Low risk	Low risk	Low risk	Unclear risk
Deng 2017	Unclear risk	Unclear risk	Unclear risk	Unclear risk	Low risk	Low risk	Low risk	Unclear risk
Zhao 2016	Unclear risk	Unclear risk	Unclear risk	Unclear risk	Low risk	Low risk	Low risk	Unclear risk
Tan 2016	Unclear risk	Unclear risk	Unclear risk	Unclear risk	Low risk	Low risk	Low risk	Unclear risk
Guo 2016a	Unclear risk	Unclear risk	Unclear risk	Unclear risk	Low risk	Low risk	Low risk	Unclear risk
Zhu 2015	Unclear risk	Unclear risk	Unclear risk	Unclear risk	Low risk	Low risk	Low risk	Unclear risk
Zhao 2015	Unclear risk	Unclear risk	Unclear risk	Unclear risk	Low risk	Low risk	Low risk	Unclear risk
Zhang 2015	Low risk	Unclear risk	Unclear risk	Unclear risk	Low risk	Low risk	Low risk	Unclear risk
Ye 2015	Unclear risk	Unclear risk	Unclear risk	Unclear risk	Low risk	Low risk	Low risk	Unclear risk
Yang 2015	Unclear risk	Unclear risk	Unclear risk	Unclear risk	Low risk	Low risk	Low risk	Unclear risk
Xia 2015	Low risk	Unclear risk	Unclear risk	Unclear risk	Low risk	Low risk	Low risk	Unclear risk
Wei 2015	Unclear risk	Unclear risk	Unclear risk	Unclear risk	Low risk	Low risk	Low risk	Unclear risk
Peng 2015	Unclear risk	Unclear risk	Unclear risk	Unclear risk	Low risk	Low risk	Low risk	Unclear risk
Liu 2015	Unclear risk	Unclear risk	Unclear risk	Unclear risk	Low risk	Low risk	Low risk	Unclear risk

Hu 2015a	Unclear risk	Unclear risk	Unclear risk	Unclear risk	Low risk	Low risk	Low risk	Unclear risk
Chen 2015	Unclear risk	Unclear risk	Unclear risk	Unclear risk	Low risk	Low risk	Low risk	Unclear risk
Yang 2014a	Low risk	Unclear risk	Unclear risk	Unclear risk	Low risk	Low risk	Low risk	Unclear risk
Yang 2014b	Unclear risk	Unclear risk	Unclear risk	Unclear risk	Low risk	Low risk	Low risk	Unclear risk
Xiao 2014	Unclear risk	Unclear risk	Unclear risk	Unclear risk	Low risk	Low risk	Low risk	Unclear risk
Tong 2014	Unclear risk	Unclear risk	Unclear risk	Unclear risk	Low risk	Low risk	Low risk	Unclear risk
Guo 2014	Low risk	Unclear risk	Unclear risk	Unclear risk	Low risk	Low risk	Low risk	Unclear risk
Bu 2014	Unclear risk	Unclear risk	Unclear risk	Unclear risk	Low risk	Low risk	Low risk	Unclear risk
Zhuo 2013	Unclear risk	Unclear risk	Unclear risk	Unclear risk	Low risk	Low risk	Low risk	Unclear risk
Zhang 2013a	Unclear risk	Unclear risk	Unclear risk	Unclear risk	Low risk	Low risk	Low risk	Unclear risk
Xia 2013	Unclear risk	Unclear risk	Unclear risk	Unclear risk	Low risk	Low risk	Unclear risk	Unclear risk
Wen 2013	Unclear risk	Unclear risk	Unclear risk	Unclear risk	Low risk	Low risk	Low risk	Unclear risk
Wei 2013	Unclear risk	Unclear risk	Unclear risk	Unclear risk	Low risk	Low risk	Low risk	Unclear risk
Wang 2013a	Low risk	Unclear risk	Unclear risk	Unclear risk	Low risk	Low risk	Low risk	Unclear risk
Wang 2013b	Low risk	Unclear risk	Unclear risk	Unclear risk	Low risk	Low risk	Low risk	Unclear risk
Wang 2013c	Unclear risk	Unclear risk	Unclear risk	Unclear risk	Low risk	Low risk	Low risk	Unclear risk
She 2013	Unclear risk	Unclear risk	Unclear risk	Unclear risk	Low risk	Low risk	Low risk	Unclear risk
Qiu 2013	Low risk	Unclear risk	Unclear risk	Unclear risk	Low risk	Low risk	Low risk	Unclear risk
Mo 2013	Unclear risk	Unclear risk	Unclear risk	Unclear risk	Low risk	Low risk	Low risk	Unclear risk
Ma 2013	Unclear risk	Unclear risk	Unclear risk	Unclear risk	Low risk	Low risk	Low risk	Unclear risk
Gu 2013	Unclear risk	Unclear risk	Unclear risk	Unclear risk	Low risk	Low risk	Low risk	Unclear risk
Gao 2013	Unclear risk	Unclear risk	Unclear risk	Unclear risk	Low risk	Low risk	Low risk	Unclear risk
Du 2013	Unclear risk	Unclear risk	Unclear risk	Unclear risk	Low risk	Low risk	Low risk	Unclear risk

Chen 2013a	Unclear risk	Unclear risk	Low risk	Unclear risk	Low risk	Low risk	Low risk	Unclear risk
Chen 2013b	Unclear risk	Unclear risk	Unclear risk	Unclear risk	Low risk	Low risk	Low risk	Unclear risk
Zhu 2012	Unclear risk	Unclear risk	Unclear risk	Unclear risk	Low risk	Low risk	Low risk	Unclear risk
Zhou 2012a	Unclear risk	Unclear risk	Unclear risk	Unclear risk	Low risk	Low risk	Low risk	Unclear risk
Yang 2012a	Unclear risk	Unclear risk	Unclear risk	Unclear risk	Low risk	Low risk	Low risk	Unclear risk
Wu 2012	Low risk	Unclear risk	Unclear risk	Unclear risk	Low risk	Low risk	Low risk	Unclear risk
Tian 2012	Unclear risk	Unclear risk	Unclear risk	Unclear risk	Low risk	Low risk	Low risk	Unclear risk
Li 2022	Unclear risk	Unclear risk	Unclear risk	Unclear risk	Low risk	Low risk	Low risk	Unclear risk
Zhu 2021a	Low risk	Unclear risk	Unclear risk	Unclear risk	Low risk	Low risk	Low risk	Unclear risk
Zhu 2021b	Low risk	Unclear risk	Unclear risk	Unclear risk	Low risk	Low risk	Low risk	Unclear risk
Zhao 2021	Low risk	Unclear risk	Unclear risk	Unclear risk	Low risk	Low risk	Low risk	Unclear risk
Xu 2021	Unclear risk	Unclear risk	Unclear risk	Unclear risk	Low risk	Low risk	Low risk	Unclear risk
Wang 2021	Low risk	Unclear risk	Unclear risk	Unclear risk	Low risk	Low risk	Low risk	Unclear risk
She 2020	Low risk	Unclear risk	Unclear risk	Unclear risk	Low risk	Low risk	Low risk	Unclear risk
Qu 2020	Low risk	Unclear risk	Unclear risk	Unclear risk	Low risk	Low risk	Low risk	Unclear risk
He 2020	Low risk	Unclear risk	Unclear risk	Unclear risk	Low risk	Low risk	Low risk	Unclear risk
Cen 2020	Low risk	Unclear risk	Unclear risk	Unclear risk	Low risk	Low risk	Low risk	Unclear risk
Cai 2020	Low risk	Unclear risk	Unclear risk	Unclear risk	Low risk	Low risk	Low risk	Unclear risk
Zhao 2019	Low risk	Unclear risk	Unclear risk	Unclear risk	Low risk	Low risk	Low risk	Unclear risk
Zhang 2019a	Low risk	Unclear risk	Unclear risk	Unclear risk	Low risk	Low risk	Low risk	Unclear risk
Zhang 2019b	Low risk	Unclear risk	Unclear risk	Unclear risk	Low risk	Low risk	Low risk	Unclear risk
Lin 2019	Low risk	Unclear risk	Unclear risk	Unclear risk	Low risk	Low risk	Low risk	Unclear risk
Bi 2019	Unclear risk	Unclear risk	Unclear risk	Unclear risk	Low risk	Low risk	Low risk	Unclear risk

Bai 2019	Unclear risk	Unclear risk	Unclear risk	Unclear risk	Low risk	Low risk	Low risk	Unclear risk
Zhou 2018	Low risk	Unclear risk	Unclear risk	Unclear risk	Low risk	Low risk	Low risk	Unclear risk
Zhao 2018	Unclear risk	Unclear risk	Unclear risk	Unclear risk	Low risk	Low risk	Low risk	Unclear risk
Zhang 2018b	Unclear risk	Unclear risk	Unclear risk	Unclear risk	Low risk	Low risk	Low risk	Unclear risk
Xu 2018	Unclear risk	Unclear risk	Unclear risk	Unclear risk	Low risk	Low risk	Low risk	Unclear risk
Wang 2018b	Low risk	Unclear risk	Unclear risk	Unclear risk	Low risk	Low risk	Low risk	Unclear risk
Tian 2018	Low risk	Unclear risk	Unclear risk	Unclear risk	Low risk	Low risk	Low risk	Unclear risk
Lin 2018	Unclear risk	Unclear risk	Unclear risk	Unclear risk	Low risk	Low risk	Low risk	Unclear risk
Li 2018	Unclear risk	Unclear risk	Unclear risk	Unclear risk	Low risk	Low risk	Low risk	Unclear risk
Yuan 2017	Unclear risk	Unclear risk	Unclear risk	Unclear risk	Low risk	Low risk	Low risk	Unclear risk
Tan 2017	Low risk	Unclear risk	Unclear risk	Unclear risk	Low risk	Low risk	Low risk	Unclear risk
Lu 2017	Low risk	Unclear risk	Unclear risk	Unclear risk	Low risk	Low risk	Low risk	Unclear risk
Liu 2017b	Unclear risk	Unclear risk	Unclear risk	Unclear risk	Low risk	Low risk	Low risk	Unclear risk
Liang 2017b	Unclear risk	Unclear risk	Unclear risk	Unclear risk	Low risk	Low risk	Low risk	Unclear risk
Huang 2017	Unclear risk	Unclear risk	Unclear risk	Unclear risk	Low risk	Low risk	Low risk	Unclear risk
Hu 2017	Low risk	Unclear risk	Unclear risk	Unclear risk	Low risk	Low risk	Low risk	Unclear risk
Zhou 2016	Unclear risk	Unclear risk	Unclear risk	Unclear risk	Low risk	Low risk	Low risk	Unclear risk
Shao 2016	Unclear risk	Unclear risk	Unclear risk	Unclear risk	Low risk	Low risk	Low risk	Unclear risk
Ma 2016	Low risk	Unclear risk	Unclear risk	Unclear risk	Low risk	Low risk	Low risk	Unclear risk
Guo 2016b	Unclear risk	Unclear risk	Unclear risk	Unclear risk	Low risk	Low risk	Low risk	Unclear risk
Deng 2016	Unclear risk	Unclear risk	Unclear risk	Unclear risk	Low risk	Low risk	Low risk	Unclear risk
Cai 2016	Unclear risk	Unclear risk	Unclear risk	Unclear risk	Low risk	Low risk	Low risk	Unclear risk
Yan 2015	Unclear risk	Unclear risk	Unclear risk	Unclear risk	Low risk	Low risk	Low risk	Unclear risk

Xie 2015	Low risk	Unclear risk	Unclear risk	Unclear risk	Low risk	Low risk	Low risk	Unclear risk
Wang 2015	Unclear risk	Unclear risk	Unclear risk	Unclear risk	Low risk	Low risk	Low risk	Unclear risk
Sun 2015	Unclear risk	Unclear risk	Unclear risk	Unclear risk	Low risk	Low risk	Low risk	Unclear risk
Lin 2015	Unclear risk	Unclear risk	Unclear risk	Unclear risk	Low risk	Low risk	Low risk	Unclear risk
Hu 2015b	Unclear risk	Unclear risk	Unclear risk	Unclear risk	Low risk	Unclear risk	Unclear risk	Unclear risk
Feng 2015	Unclear risk	Unclear risk	Unclear risk	Unclear risk	Low risk	Low risk	Low risk	Unclear risk
Duan 2015	Low risk	Unclear risk	Unclear risk	Unclear risk	Low risk	Low risk	Low risk	Unclear risk
Zhao 2014	Unclear risk	Unclear risk	Unclear risk	Unclear risk	Low risk	Low risk	Low risk	Unclear risk
Xu 2014	Unclear risk	Unclear risk	Unclear risk	Unclear risk	Low risk	Low risk	Low risk	Unclear risk
Shen 2014	Unclear risk	Unclear risk	Unclear risk	Unclear risk	Low risk	Low risk	Low risk	Unclear risk
Peng 2014	Unclear risk	Unclear risk	Unclear risk	Unclear risk	Low risk	Low risk	Low risk	Unclear risk
Miao 2014	Unclear risk	Unclear risk	Unclear risk	Unclear risk	Low risk	Low risk	Low risk	Unclear risk
Ling 2014	Unclear risk	Unclear risk	Unclear risk	Unclear risk	Low risk	Low risk	Low risk	Unclear risk
Li 2014a	Unclear risk	Unclear risk	Unclear risk	Unclear risk	Low risk	Low risk	Low risk	Unclear risk
Li 2014b	Unclear risk	Unclear risk	Unclear risk	Unclear risk	Low risk	Low risk	Low risk	Unclear risk
Guan 2014	Low risk	Unclear risk	Unclear risk	Unclear risk	Low risk	Low risk	Low risk	Unclear risk
Chen 2014	Unclear risk	Unclear risk	Unclear risk	Unclear risk	Low risk	Low risk	Low risk	Unclear risk
Zhou 2013a	Unclear risk	Unclear risk	Unclear risk	Unclear risk	Low risk	Unclear risk	Unclear risk	Unclear risk
Zhou 2013b	Unclear risk	Unclear risk	Unclear risk	Unclear risk	Low risk	Low risk	Low risk	Unclear risk
Zhao 2013	Unclear risk	Unclear risk	Unclear risk	Unclear risk	Low risk	Low risk	Low risk	Unclear risk
Zhang 2013b	Unclear risk	Unclear risk	Unclear risk	Unclear risk	Low risk	Low risk	Low risk	Unclear risk
Wang 2013d	Unclear risk	Unclear risk	Unclear risk	Unclear risk	Low risk	Low risk	Low risk	Unclear risk
Luo 2013	Unclear risk	Unclear risk	Unclear risk	Unclear risk	Low risk	Low risk	Low risk	Unclear risk

Jia 2013a	Unclear risk	Unclear risk	Unclear risk	Unclear risk	Low risk	Low risk	Low risk	Unclear risk
Jia 2013b	Unclear risk	Unclear risk	Unclear risk	Unclear risk	Low risk	Low risk	Low risk	Unclear risk
Hou 2013	Unclear risk	Unclear risk	Unclear risk	Unclear risk	Low risk	Low risk	Low risk	Unclear risk
Guo 2013	Low risk	Unclear risk	Unclear risk	Unclear risk	Low risk	Low risk	Low risk	Unclear risk
Fang 2013	Unclear risk	Unclear risk	Unclear risk	Unclear risk	Low risk	Low risk	Low risk	Unclear risk
Zhou 2012b	Unclear risk	Unclear risk	Unclear risk	Unclear risk	Low risk	Low risk	Low risk	Unclear risk
Zhang 2012	Unclear risk	Unclear risk	Unclear risk	Unclear risk	Low risk	Low risk	Low risk	Unclear risk
Yin 2012	Unclear risk	Unclear risk	Unclear risk	Unclear risk	Low risk	Low risk	Low risk	Unclear risk
Yi 2012	Unclear risk	Unclear risk	Unclear risk	Unclear risk	Low risk	Low risk	Low risk	Unclear risk
Yang 2012b	Unclear risk	Unclear risk	Unclear risk	Unclear risk	Low risk	Low risk	Low risk	Unclear risk
Yang 2012c	Unclear risk	Unclear risk	Unclear risk	Unclear risk	Low risk	Low risk	Low risk	Unclear risk
Tang 2012	Unclear risk	Unclear risk	Unclear risk	Unclear risk	Low risk	Low risk	Low risk	Unclear risk
Song 2012	Low risk	Unclear risk	Unclear risk	Unclear risk	Low risk	Low risk	Low risk	Unclear risk
Meng 2012	Unclear risk	Unclear risk	Unclear risk	Unclear risk	Low risk	Low risk	Low risk	Unclear risk
Ma 2012	Unclear risk	Unclear risk	Unclear risk	Unclear risk	Low risk	Low risk	Low risk	Unclear risk
Liu 2012a	Unclear risk	Unclear risk	Unclear risk	Unclear risk	Low risk	Low risk	Low risk	Unclear risk
LIu 2012b	Unclear risk	Unclear risk	Unclear risk	Unclear risk	Low risk	Low risk	Low risk	Unclear risk
Liu 2012c	Unclear risk	Unclear risk	Unclear risk	Unclear risk	Low risk	Low risk	Low risk	Unclear risk
Liang 2012	Unclear risk	Unclear risk	Unclear risk	Unclear risk	Low risk	Unclear risk	Unclear risk	Unclear risk
Li 2012	Unclear risk	Unclear risk	Unclear risk	Unclear risk	Low risk	Low risk	Low risk	Unclear risk
Jiao 2012	Unclear risk	Unclear risk	Unclear risk	Unclear risk	Low risk	Low risk	Low risk	Unclear risk
Huang 2012	Low risk	Unclear risk	Unclear risk	Unclear risk	Low risk	Low risk	Low risk	Unclear risk
Guo 2012	Unclear risk	Unclear risk	Unclear risk	Unclear risk	Low risk	Low risk	Low risk	Unclear risk

Gong 2012	Unclear risk	Unclear risk	Unclear risk	Unclear risk	Low risk	Low risk	Low risk	Unclear risk
Fu 2012	Unclear risk	Unclear risk	Unclear risk	Unclear risk	Low risk	Low risk	Low risk	Unclear risk
Zhang 2011	Unclear risk	Unclear risk	Unclear risk	Unclear risk	Low risk	Low risk	Low risk	Unclear risk
Yu 2011	Low risk	Unclear risk	Unclear risk	Unclear risk	Low risk	Low risk	Low risk	Unclear risk
Wang 2011	Unclear risk	Unclear risk	Unclear risk	Unclear risk	Low risk	Low risk	Low risk	Unclear risk
Peng 2011	Unclear risk	Unclear risk	Unclear risk	Unclear risk	Low risk	Low risk	Low risk	Unclear risk
Mao 2011	Unclear risk	Unclear risk	Unclear risk	Unclear risk	Low risk	Low risk	Low risk	Unclear risk
Liu 2011	Unclear risk	Unclear risk	Unclear risk	Unclear risk	Low risk	Low risk	Low risk	Unclear risk
Leng 2011	Unclear risk	Unclear risk	Unclear risk	Unclear risk	Low risk	Low risk	Low risk	Unclear risk
Guo 2011	Unclear risk	Unclear risk	Unclear risk	Unclear risk	Low risk	Low risk	Low risk	Unclear risk
Chen 2011	Low risk	Unclear risk	Unclear risk	Unclear risk	Low risk	Low risk	Low risk	Unclear risk
Wang 2010a	Unclear risk	Unclear risk	Unclear risk	Unclear risk	Low risk	Low risk	Low risk	Unclear risk
Wang 2010b	Unclear risk	Unclear risk	Unclear risk	Unclear risk	Low risk	Low risk	Low risk	Unclear risk
Lu 2010	Unclear risk	Unclear risk	Unclear risk	Unclear risk	Low risk	Low risk	Low risk	Unclear risk
Li 2010a	Unclear risk	Unclear risk	Unclear risk	Unclear risk	Low risk	Low risk	Low risk	Unclear risk
Li 2010b	Unclear risk	Unclear risk	Unclear risk	Unclear risk	Low risk	Low risk	Low risk	Unclear risk
Gao 2010	Unclear risk	Unclear risk	Unclear risk	Unclear risk	Low risk	Low risk	Low risk	Unclear risk
Chen 2010	Unclear risk	Unclear risk	Unclear risk	Unclear risk	Low risk	Low risk	Low risk	Unclear risk
Cao 2010	Unclear risk	Unclear risk	Unclear risk	Unclear risk	Low risk	Low risk	Low risk	Unclear risk
Zhou 2009	Unclear risk	Unclear risk	Unclear risk	Unclear risk	Low risk	Low risk	Low risk	Unclear risk
Zhang 2009	Unclear risk	Unclear risk	Unclear risk	Unclear risk	Low risk	Low risk	Low risk	Unclear risk
Wang 2009	Unclear risk	Unclear risk	Unclear risk	Unclear risk	Low risk	Low risk	Low risk	Unclear risk
Mei 2009	Unclear risk	Unclear risk	Unclear risk	Unclear risk	Low risk	Low risk	Low risk	Unclear risk

Chen 2009	Unclear risk	Unclear risk	Unclear risk	Unclear risk	Low risk	Low risk	Low risk	Unclear risk
Zhen 2007	Unclear risk	Unclear risk	Unclear risk	Unclear risk	Low risk	Low risk	Low risk	Unclear risk
Xu 2007b	Unclear risk	Unclear risk	Unclear risk	Unclear risk	Low risk	Low risk	Low risk	Unclear risk
Ma 2007	Unclear risk	Unclear risk	Unclear risk	Unclear risk	Low risk	Low risk	Low risk	Unclear risk

Supplementary 4 Meta-analysis results

Table S4 Risk of bias of 14 randomised controlled trials included in meta-analysis

Study ID	Random sequence generation (selection bias)	Allocation concealment (selection bias)	Blinding of participants and personnel (performance bias)	Blinding of outcome assessment (detection bias)	Incomplete outcome data (attrition bias)	Selective reporting (reporting bias)	Other bias	Overall
Zhou 2008	Unclear risk	Unclear risk	Unclear risk	Unclear risk	Low risk	Low risk	Low risk	Unclear risk
Wang 2008	Unclear risk	Unclear risk	Unclear risk	Unclear risk	Low risk	Low risk	Low risk	Unclear risk
Long 2008	Unclear risk	Unclear risk	Unclear risk	Unclear risk	Low risk	Low risk	Low risk	Unclear risk
Wang 2006	Unclear risk	Unclear risk	Unclear risk	Unclear risk	Low risk	Low risk	Low risk	Unclear risk
Huang 2005	Unclear risk	Unclear risk	Unclear risk	Unclear risk	Low risk	Low risk	Low risk	Unclear risk
Zhao 2014	Unclear risk	Unclear risk	Unclear risk	Unclear risk	Low risk	Low risk	Low risk	Unclear risk
Hou 2013	Unclear risk	Unclear risk	Unclear risk	Unclear risk	Low risk	Low risk	Low risk	Unclear risk
Fang 2013	Unclear risk	Unclear risk	Unclear risk	Unclear risk	Low risk	Low risk	Low risk	Unclear risk
Zhang 2012	Unclear risk	Unclear risk	Unclear risk	Unclear risk	Low risk	Low risk	Low risk	Unclear risk
Yu 2011	Low risk	Unclear risk	Unclear risk	Unclear risk	Low risk	Low risk	Low risk	Unclear risk
Lu 2010	Unclear risk	Unclear risk	Unclear risk	Unclear risk	Low risk	Low risk	Low risk	Unclear risk
Gao 2010	Unclear risk	Unclear risk	Unclear risk	Unclear risk	Low risk	Low risk	Low risk	Unclear risk
Wang 2009	Unclear risk	Unclear risk	Unclear risk	Unclear risk	Low risk	Low risk	Low risk	Unclear risk
Ma 2007	Unclear risk	Unclear risk	Unclear risk	Unclear risk	Low risk	Low risk	Low risk	Unclear risk
